# Pan-genome de Bruijn graph using the bidirectional FM-index

**DOI:** 10.1186/s12859-023-05531-6

**Published:** 2023-10-26

**Authors:** Lore Depuydt, Luca Renders, Thomas Abeel, Jan Fostier

**Affiliations:** 1 Department of Information Technology - IDLab, Ghent University - imec, Technologiepark 126, 9052 Ghent, Belgium; 2 Delft Bioinformatics Lab, Delft University of Technology, 2628 XE Delft, The Netherlands; 3 Infectious Disease and Microbiome Program, Broad Institute of MIT and Harvard, Cambridge, MA 02142 USA

**Keywords:** Approximate pattern matching, Sequence-to-graph alignment, Search schemes, Lossless alignment, Pan-genome visualization

## Abstract

**Background:**

Pan-genome graphs are gaining importance in the field of bioinformatics as data structures to represent and jointly analyze multiple genomes. Compacted de Bruijn graphs are inherently suited for this purpose, as their graph topology naturally reveals similarity and divergence within the pan-genome. Most state-of-the-art pan-genome graphs are represented explicitly in terms of nodes and edges. Recently, an alternative, implicit graph representation was proposed that builds directly upon the unidirectional FM-index. As such, a memory-efficient graph data structure is obtained that inherits the FM-index’ backward search functionality. However, this representation suffers from a number of shortcomings in terms of functionality and algorithmic performance.

**Results:**

We present a data structure for a pan-genome, compacted de Bruijn graph that aims to address these shortcomings. It is built on the bidirectional FM-index, extending the ability of its unidirectional counterpart to navigate and search the graph in both directions. All basic graph navigation steps can be performed in constant time. Based on these features, we implement subgraph visualization as well as lossless approximate pattern matching to the graph using search schemes. We demonstrate that we can retrieve all occurrences corresponding to a read within a certain edit distance in a very efficient manner. Through a case study, we show the potential of exploiting the information embedded in the graph’s topology through visualization and sequence alignment.

**Conclusions:**

We propose a memory-efficient representation of the pan-genome graph that supports subgraph visualization and lossless approximate pattern matching of reads against the graph using search schemes. The C++ source code of our software, called Nexus, is available at https://github.com/biointec/nexus under AGPL-3.0 license.

**Supplementary Information:**

The online version contains supplementary material available at 10.1186/s12859-023-05531-6.

## Background

Modern sequencing platforms enable the rapid sequencing of genomes. Whereas one consensus reference genome per species used to be the norm, it is now common to have thousands of genomes for a single species. New techniques must be developed to efficiently store, manipulate, analyze and visualize large genomic collections (often representing a species or clade). These collections, analyzed jointly or used as a reference, are referred to as pan-genomes [1, 2]. A key innovation in pan-genomics is the adoption of graphs as the primary form of representation, as graphs are inherently suited to summarize multiple genomes into a single data structure by compacting shared regions into common nodes. As such, pan-genome graphs can robustly and intuitively encode natural variation, such as SNPs and structural variation [3].

Pan-genome graphs can be sequence-based, gene-based, or a combination. Sequence-based pan-genome graphs consist of nodes representing sequences and edges denoting adjacencies between them. They are ideal for detailed analysis of highly similar input genomes, such as human individuals. On the other hand, gene-based approaches (distinguishing core genes, dispensable genes and strain-specific genes [4]) are more suitable for pan-genomes of distantly related organisms with less conserved sequence content. This paper focuses on sequence-based pan-genomes.

The emergence of pan-genome graphs has enabled various functionalities [2]. Existing bioinformatics analyses relying on a reference genome are often biased towards the specific choice of reference [5–9]. Since pan-genome graphs can mitigate this reference bias, the Computational Pan-Genomics Consortium proposes the following design goal: “Comparisons of short and long sequences (e.g. reads) with the pan-genome ideally results in the *corresponding location* and the *best matching individual genome(s)*” [2]. Pan-genome graphs also facilitate knowledge extraction through topological analysis [3], revealing (the degree of) similarity between the input genomes, the presence of (structural) variation, conserved regions, etc. Visualization of the graph enables the investigation of these features, which is why “all information within the data structure should be easily accessible for human eyes by visualization support on different scales” [2].

### State-of-the-art pan-genome representations

The most straightforward approach for storing a pan-genome is creating a linear full-text index of the concatenated genomes. This approach offers advantages such as efficient storage and alignment using state-of-the-art linear aligners like BWA-MEM [10] and Bowtie 2 [11] (both based on the FM-index [12]), while preserving linkage disequilibrium during alignment. However, downsides include the lack of insight into the pan-genome’s characteristics and index growth proportional to the sequence-content in the pan-genome (although recent developments and implementations regarding the *r*-index [13] might alleviate this issue).

A second prominent form of pan-genome representation is a variation graph, obtained by augmenting a linear reference genome with known variation in the population. We distinguish acyclic variation graphs and general variation graphs. Some tools support only acyclic variation graphs [14–19], lacking representation of complex variations like copy number variations, inversions, and translocations. In contrast, the most popular sequence-to-graph aligners [20–23] handle general variation graphs. Variation graphs can space-efficiently incorporate variation across *many* individuals and enable the exploration of the graph topology through visualization. However, they depend on the reference genome that serves as the backbone of the graph, sequence-to-graph alignment is complex [24], and chimeric alignments can occur when isolated variations are added to the graph without preserving linkage disequilibrium. Giraffe [21] mitigates the latter by including haplotype information.

A third pan-genome representation is the de Bruijn graph (dBG), consisting of nodes representing each distinct *k*-mer in the pan-genome (i.e., the collection of all *complete* genomes) and edges connecting corresponding nodes for each (*k* + 1)-mer. Linear chains of nodes are often merged to create a compacted dBG (cdBG) with a more interpretable topology, where nodes represent unitigs and edges indicate divergence [3, 25]. Colored cdBGs (ccdBGs) assign colors to nodes and edges based on the underlying strains in which they occur [26]. Several tools construct *assembly* (cc)dBGs, and some can also perform (pseudo)alignment to them [27–35]. However, since assembly dBGs are created from a set of input reads, there is no functionality to maintain the connection between the graph (nodes) and (the coordinates of) the underlying input sequences. Therefore, these data structures and algorithms are not suitable for our problem, and vice versa. Tools that align reads to *pan-genome* dBGs and can link graph nodes back to genome coordinates are relatively scarce. Examples include deBGA [36] for dBGs and PuffAligner [37] for ccdBGs (based on the Pufferfish index [38]). However, these tools only report coordinates without providing alignment information inside the graph (i.e., node paths), and lack support for visualizing regions of interest within the graph.

Beller and Ohlebusch [39] recently proposed a memory-efficient, implicit representation of a ccdBG, built upon the unidirectional FM-index of the underlying sequences. The graph edges are not explicitly stored; instead, the FM-index and a few additional arrays enable graph navigation. The FM-index also allows for pattern matching against the graph. However, the current implementation is limited to exact pattern matching, while approximate pattern matching (APM) is more relevant for bioinformatics applications due to sequencing errors and genetic variation. Also, only backward traversal of the graph is supported due to the underlying unidirectional FM-index, restricting visualization to asymmetric subgraphs (i.e., only the upstream neighborhood of the node(s) of interest) [40]. Finally, identifying a node containing a specific *k*-mer is an *O*(*n*) operation (with *n* the size of the pan-genome), which can be slow in practice. This paper aims to address these limitations.

### Contributions

Inspired by the work of Beller and Ohlebusch, we propose a memory-efficient, colored, compacted de Bruijn Graph (ccdBG) representation that is built upon the *bidirectional* FM-index [41]. Specifically, we make the following contributions:

(i) Leveraging the bidirectional FM-index, our graph representation supports *bidirectional* (i.e., forward and backward) navigation of the graph in O(1) time per step. Implementing this functionality in implicit graph representations is non-trivial. Additionally, we present an algorithm for visualizing a region of interest with its complete neighborhood, generating symmetric subgraphs.
(ii) Our graph representation is built upon the bidirectional FM-index in a modular manner, allowing seamless integration of advancements for the bidirectional FM-index into our pan-genome graph. We demonstrate this by applying *search schemes* [42] to enable efficient lossless approximate pattern matching against our pan-genome graph under the edit distance metric (allowing substitutions and indels). Search schemes are a class of sequence alignment algorithms that, using a bidirectional full-text index, prioritize quick elimination of unsuccessful search branches to minimize runtime. Their excellent performance has been demonstrated for linear reference genomes [42–45]. Unlike lossy heuristics (often relying on the seed-and-extend paradigm), search schemes are lossless: they guarantee to retrieve *all* occurrences within a specified error distance. As pan-genome graphs can comprise hundreds of similar sequences (e.g., closely related bacterial strains), lossless algorithms that efficiently report all occurrences appear particularly attractive. As outputs, we report occurrences both as walks in the graph and as coordinates within the underlying sequences.
(iii) We introduce checkpoint *k*-mers to reduce the time complexity to identify the graph node corresponding to a given *k*-mer from O(n) to O(1) (with *n* the size of the pan-genome). In practice, this results in a significant speedup, with the node path identification step being up to 3 times faster. This improvement comes at a minimal additional memory cost.

This paper is organized as follows. We first describe the data structure with its support for graph navigation in constant-time, subgraph visualization, and efficient lossless approximate pattern matching using search schemes. In the results section, we demonstrate the functionalities and performance of our tool. We show that the graph representation requires far less memory than the underlying bidirectional FM-index. We analyze the performance of our approximate pattern matching implementation, comparing it with other tools and exploring the impact of the checkpoint sparseness factor. We present a case study on a *Mycobacterium tuberculosis* pan-genome to illustrate the extraction of information from the graph topology.

## Methods

### Preliminaries

Zero-based indexing is used for strings and arrays. Consider a text *T* of length $$n=|T|$$ over alphabet $$\Sigma$$. In a pan-genome context, *T* is the concatenation of multiple DNA sequences, separated by ‘%’ characters. We denote the number of sequences in *T* by *S*. The sentinel character ‘$’, a unique character lexicographically smaller than any other character in $$\Sigma$$, is appended to *T*. Character ‘%’ is the lexicographically second smallest character in $$\Sigma$$. Characters ‘%’ and ‘$’ are referred to as separation characters. A substring of a string *T* is denoted by a half-open interval *T*[*i*, *j*[, with $$0\le i\le j \le n$$. The *i*th suffix of *T*, denoted as $$T_i$$, is the substring *T*[*i*, *n*[. Analogously, substring *T*[0, *i*[ is the *i*th prefix of *T*.

A de Bruijn graph (dBG) G(*V*, *E*) [46] is a directed graph where the nodes are all *k*-mers (i.e., *k*-length substrings) present in *T*. We omit *k*-mers that contain a separation character (‘%’, ‘$’) in any but their last position. A directed edge connects two nodes *u* and *v* when a $$(k+1)$$-mer exists in *T* for which the first *k* nucleotides coincide with *u* and the last *k* nucleotides coincide with *v*. If multiple such $$(k+1)$$-mers exist in *T*, we draw the corresponding number of edges between nodes *u* and *v*. In other words, G(*V*, *E*) is a multigraph. Note that we do not create a bidirected genome graph, i.e., a *k*-mer and its reverse complement are not represented by the same node. A *compacted* de Bruijn graph (cdBG) is obtained by maximally contracting all pairs of connected nodes *u* and *v* for which *v* is the sole successor of *u* and, vice versa, *u* is the sole predecessor of *v*. Nodes of a cdBG thus represent substrings of *T* of length $$\ge k$$, referred to as *unitigs*. A *colored* compacted de Bruijn graph (ccdBG) retains the origin strain of each edge by assigning it a color.

**Fig. 1:**
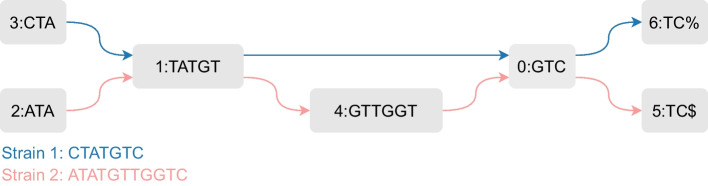
ccdBG ($$k = 3$$) for *T* = “CTATGTC%ATATGTTGGTC$”

Throughout this paper, we illustrate the data structures and algorithms using *T* = “CTATGTC%ATATGTTGGTC$” as a small example pan-genome with $$S=2$$ sequences. Figure 1 shows this example’s ccdBG ($$k=3$$).

### Bidirectional graph data structure

#### Bidirectional FM-index

Our implicit representation of the ccdBG G(*V*, *E*) is built upon the bidirectional FM-index of *T*. Readers less familiar with the bidirectional FM-index are referred to the supplementary material for a brief overview. Table 2 illustrates for our example text *T*, the corresponding suffix array SA [47], Burrows-Wheeler transform BWT [48], LF mapping and sorted suffixes. Similarly, Table 4 shows the reverse text $T^r$, its suffix array $\textrm{SA}^r$, Burrows-Wheeler transform $\textrm{BWT}^r$, LF mapping and sorted suffixes. Note that all variables related to the reverse part of the bidirectional FM-index are denoted with a superscript *r*. Bit vectors B and B^r^ will be explained later. Exact occurrences of a search pattern *P* in *T* are represented in the bidirectional FM-index by two intervals: an interval [*b*, *e*[ over SA and an interval $[b^r, e^r[$ over $\textrm{SA}^r$, such that all suffixes $T_{\textrm{SA}[i]}$ for $b \le i < e$ have *P* as their prefix while suffixes $T^r_{\textrm{SA}^r[i]}$ for $b^r \le i < e^r$ are prefixed by $P^r$, the reverse of *P*. For example, for search pattern *P* = “ATG”, $\textrm{SA}[3,5[$ refers to the suffixes of *T* prefixed by *P*, while $\textrm{SA}^r[9,11[$ refers to suffixes of $T^r$ prefixed by $P^r$ = “GTA”. Patterns are matched character by character: given a pattern *P* and its intervals [*b*, *e*[ and $[b^r,e^r[$, the intervals $[b',e'[$ and $[b{^r}',e{^r}'[$ of the extended pattern *cP* (extendBackward) or *Pc* (extendForward) can be found in $\textrm{O}(1)$ time [49]. In other words, the key functionality of a bidirectional FM-index entails that a partial match can be extended with a character either to the left or to the right.

#### Collection of graph nodes

The data corresponding to the nodes of the ccdBG is stored in a vector G of length |*V*|, with |*V*| the number of nodes (see Table 1). Each node is assigned a unique identifier $id \in \{0,\ldots ,|V|-1\}$. This way, the node with identifier $id$ can be accessed at $\textrm{G}[ id ]$. Each node represents a substring $\omega$ of *T*. This substring is not explicitly stored in G. Every node has four attributes: $len$, $mult$, $left\_kmer$, and $right\_kmer^r$. Here, $len$ denotes the length of $\omega$, while $left\_kmer$ is the left boundary of the SA interval that corresponds to $\omega$. Consequently, $\omega$ can be deduced from the node attributes as $T[\textrm{SA}[ left\_kmer ],\textrm{SA}[ left\_kmer ]+ len [$. Due to the characteristics of a cdBG, $left\_kmer$ is also the left boundary of the suffix array interval that corresponds to the leftmost *k*-mer of $\omega$. The $mult$ attribute corresponds to the multiplicity of the node, which is the number of times $\omega$ occurs in *T*. Hence, $mult$ is also the size of $\omega$’s suffix array interval: $\textrm{SA}[ left\_kmer , left\_kmer + mult [$. Analogously, $right\_kmer^r$ represents for the *reverse* of the rightmost *k*-mer of a node, the left boundary of its interval in the *reverse* suffix array. Consequently, the reverse suffix array interval of the reverse rightmost *k*-mer of the node can be found as $\textrm{SA}^r[ right\_kmer^r , right\_kmer^r + mult [$. For example, consider the node with $id = 4$ in Table 1. Its leftmost *k*-mer, “GTT”, has its left boundary of the SA interval at index 11 (see Table 2). Similarly, the interval of the reverse rightmost *k*-mer “TGG” in $\textrm{SA}^r$ starts at index 16 (see Table 4).

**Table 1:** Vector G for *T* = “CTATGTC%ATATGTTGGTC$”

*id*	*len*	*mult*	*left*_*km*er	*right*_*kmer*^r^	*ω*
0	3	2	9	6	GTC
1	5	2	12	17	TATGT
2	3	1	2	3	ATA
3	3	1	7	4	CTA
4	6	1	11	16	GTTGGT
5	3	1	14	15	TC$
6	3	1	15	12	TC%

Attribute *ω* is shown only for illustration purposes and is not stored in the G vector

The end nodes form an exception to the rules defined above: their rightmost *k*-mer is obtained from the (cyclic) extension of $$\omega$$ by the next $$k-1$$ characters in *T* (e.g., “$CT” for node 5 and “%AT” for node 6 in Fig. 1). Additionally, each of the *S* sequences in *T* gets a *distinct* end node in vector G, even if they correspond to the same string $$\omega$$. For more detailed information, the reader is referred to [39].

**Table 2:** Search text $$T =$$ “CTATGTC%ATATGTTGGTC$” with its suffix array SA, Burrows-Wheeler transform BWT, bit vector B (for $$k=3$$), LF mapping and suffixes

$$i$$	$$T$$	$$SA$$	$$BWT$$	$$B$$	$$LF$$	$$T_{\textrm{SA}[i]}$$
0	C	19	C	1	5	$
1	T	7	C	1	6	%ATATGTTGGTC$
2	A	8	%	1	1	ATATGTTGGTC$
3	T	2	T	0	12	ATGTC%ATATGTTGGTC$
4	G	10	T	0	13	ATGTTGGTC$
5	T	18	T	0	14	C$
6	C	6	T	0	15	C%ATATGTTGGTC$
7	%	0	$	1	0	CTATGTC%ATATGTTGGTC$
8	A	15	T	1	16	GGTC$
9	T	16	G	0	8	GTC$
10	A	4	T	1	17	GTC%ATATGTTGGTC$
11	T	12	T	0 (1)	18	GTTGGTC$
12	G	1	C	0	7	TATGTC%ATATGTTGGTC$
13	T	9	A	0 (1)	2	TATGTTGGTC$
14	T	17	G	0	9	TC$
15	G	5	G	0	10	TC%ATATGTTGGTC$
16	G	14	T	0 (1)	19	TGGTC$
17	T	3	A	0	3	TGTC%ATATGTTGGTC$
18	C	11	A	1	4	TGTTGGTC$
19	$	13	G	0	11	TTGGTC$

The entries in parentheses will be detailed later

**Table 3:** $\textrm{IDmap}$ corresponding to Table 2

$id_{\textrm{B}}$	0	1	2	3	4	5	6
$id$	5	6	2	3	4	0	1

First row: identifiers deduced from bit vector B, second row: node identifiers

**Table 4:** Reverse search text $$T^r =$$ “$CTGGTTGTATA%CTGTATC” with its suffix array $$\textrm{SA}^r$$, Burrows-Wheeler transform $$\textrm{BWT}^r$$, bit vector $$\textrm{B}^r$$ (for $$k=3$$), LF mapping and suffixes

$$i$$	$$T^r$$	$$\textrm{SA}^r$$	$$\mathrm {BWT^r}$$	$$\textrm{B}^r$$	$$\textrm{LF}^r$$	$$T^r_{\textrm{SA}^r[i]}$$
0	$	0	C	1	5	$CTGGTTGTATA%CTGTATC
1	C	12	A	1	2	%CTGTATC
2	T	11	T	0	12	A%CTGTATC
3	G	9	T	1	13	ATA%CTGTATC
4	G	17	T	1	14	ATC
5	T	19	T	0	15	C
6	T	1	$	0	0	CTGGTTGTATA%CTGTATC
7	G	13	%	1	1	CTGTATC
8	T	3	T	0	16	GGTTGTATA%CTGTATC
9	A	7	T	0	17	GTATA%CTGTATC
10	T	15	T	0	18	GTATC
11	A	4	G	0	8	GTTGTATA%CTGTATC
12	%	10	A	0	3	TA%CTGTATC
13	C	8	G	0	9	TATA%CTGTATC
14	T	16	G	1	10	TATC
15	G	18	A	0	4	TC
16	T	2	C	0	6	TGGTTGTATA%CTGTATC
17	A	6	T	0	19	TGTATA%CTGTATC
18	T	14	C	0	7	TGTATC
19	C	5	G	1	11	TTGTATA%CTGTATC

**Table 5:** $\textrm{IDmap}^r$ corresponding to Table 4

$id_{\textrm{B}^r}$	0	1	2	3	4	5	6
$id$	5	6	2	3	0	1	4

First row: identifiers deduced from bit vector $\textrm{B}^r$, second row: node identifiers

#### Auxiliary bit vectors and tables

The bidirectional FM-index (Tables 2 and 4) is supplemented with two auxiliary bit vectors $\textrm{B}$ and $\textrm{B}^r$.

$\textrm{B}[i] = 1$ if the following two conditions apply:

1. *k*-mer $T[\textrm{SA}[i], \textrm{SA}[i]+k[$ is the *rightmost* *k*-mer of a node.
2. Suffix $T_{\textrm{SA}[i]}$ is the lexicographically largest suffix of *T* that has *k*-mer $T[\textrm{SA}[i], \textrm{SA}[i]+k[$ as a prefix.

For example, “TGT” is the rightmost *k*-mer of node 1 and is indicated by a 1-bit in $\textrm{B}$ at index 18 (Table 2). Again, for the end nodes, the rightmost *k*-mer is defined differently and each of the *S* distinct end nodes is indicated in $\textrm{B}$, even if they correspond to the same string $\omega$. Hence, the *S* first bits in $\textrm{B}$ are set to 1 for the end nodes.

Analogously, $\textrm{B}^r = 1$ if the following two conditions apply:

1. *k*-mer $T^r[\textrm{SA}^r[i], \textrm{SA}^r[i]+k[$ is the *reverse* of the *leftmost* *k*-mer of a node.
2. Suffix $T^r_{\textrm{SA}^r[i]}$ is the lexicographically largest suffix of $T^r$ that has *k*-mer $T^r[\textrm{SA}^r[i], \textrm{SA}^r[i]+k[$ as a prefix.

For example, “TTG” is the reverse of the leftmost *k*-mer of node 4 and is indicated by a 1-bit in $\textrm{B}^r$ at index 19 (Table 4).

Note that there are as many 1-bits in $\textrm{B}$ and $\textrm{B}^r$ as there are nodes in the graph. We will use bit vectors $\textrm{B}$ and $\textrm{B}^r$ to obtain node identifiers that correspond to a certain *k*-mer using rank operations. Because the 1-bits in $\textrm{B}$ and $\textrm{B}^r$ are ordered differently, we store two node identifier mappings, $\textrm{IDmap}$ and $\textrm{IDmap}^r$ (see Tables 3 and 5), which transform the rank extracted from $\textrm{B}$ and $\textrm{B}^r$ respectively, to the effective node identifier. Note that the nodes in vector $\textrm{G}$ can be ordered arbitrarily, as long as $\textrm{IDmap}$ and $\textrm{IDmap}^r$ are adjusted accordingly. Here, we choose to put the *S* end nodes at the end of vector $\textrm{G}$. This way, it can be easily assessed if a certain node identifier corresponds to an end node or not.

#### Building the data structure

The construction process of the underlying bidirectional FM-index is based on the implementation of Columba [45, 50]. The construction of components $\textrm{G}$ and $\textrm{B}$ is similar to the algorithms described in [39]. Finally, the construction of components $\textrm{B}^r$, $\textrm{IDmap}$ and $\textrm{IDmap}^r$ is a new contribution. A description of these algorithms would be quite lengthy and technical and is therefore omitted from this paper.

### Elementary graph operations

To support more complex graph operations (e.g., subgraph visualization and approximate pattern matching), we need a set of building blocks that aid in navigating the graph. We introduce three elementary graph operations:

1. Determining the node identifier given the suffix array interval of an extreme *k*-mer (i.e., the left- or rightmost *k*-mer) of that node.
2. Computing the identifier of the predecessor (resp. successor) of a given node by prepending (resp. appending) a character *c* to its substring $\omega$.
3. Obtaining the identifier of the predecessor of a node by following a specific edge, i.e., by extending a specific occurrence of $\omega$ in *T*.

#### Determining the node identifier for an extreme k-mer

Determining a node’s identifier based on its rightmost *k*-mer is important in the following scenario. If a partial match in the graph is extended with a character to the left and, as a consequence, a new node is visited, its rightmost *k*-mer is encountered first. For this partial match, the corresponding intervals [*b*, *e*[ and $[b^r, e^r[$ over $\textrm{SA}$ and $\textrm{SA}^r$ respectively, are kept track of by the bidirectional FM-index. All suffixes in interval [*b*, *e*[ then start with the rightmost *k*-mer of the new node. This node identifier can then be retrieved using function findIDRight (see Algorithm 1). Value $id_{\textrm{B}}$ is obtained by a rank operation on bit vector $\textrm{B}$ at index *b* that returns the total number of 1-bits in $\textrm{B}[0, b[$ (i.e., *before* index *b*). Next, array $\textrm{IDmap}$ maps value $id_{\textrm{B}}$ to the actual node identifier *id* which can then be used to access vector $\textrm{G}$. Assuming constant-time rank support on bit vectors [51], function findIDRight runs in $\textrm{O}(1)$ time. For example, (rightmost) *k*-mer “GTC” with interval $\textrm{SA}[9,11[$ yields $id_{\textrm{B}} = 5$ (Table 2). Node identifier $id=0$ can be found at index 5 in $\textrm{IDmap}$ (Table 3).
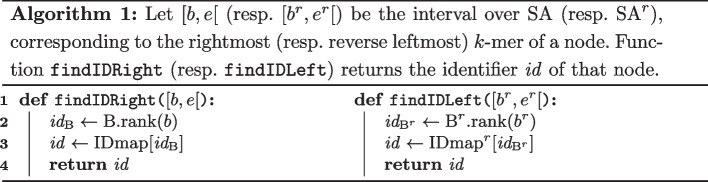


Analogously, $\textrm{B}^r$ plays an important role when matching in the forward direction, as it stores information about the leftmost *k*-mer of each node. When extending a partial match with a character to the right and a new node is visited as a consequence, its node identifier can be found using function findIDLeft in $\textrm{O}(1)$ time. For example, (reverse leftmost) *k*-mer “TAT” with interval $\textrm{SA}^r[13,15[$ yields $id_{\textrm{B}^r} = 5$ (Table 4). Node identifier $id =1$ can be found at index 5 in $\textrm{IDmap}^r$ (Table 5).

Note that for functions findIDRight and findIDLeft, it is not mandatory that the input intervals contain *all* suffixes that are prefixed by the *k*-mer of interest. In fact, the rank operation on line 2 can be called using *any* index in $\textrm{SA}$ (resp. $\textrm{SA}^r$), corresponding to the *k*-mer of interest.

#### Jumping to a neighbor with a character

Given a node identifier $id$ and a character *c*, function getPredIDWithChar computes the identifier of the predecessor node that is encountered by prepending *c* to substring $\omega$ of node $id$ (see Algorithm 2). On line 3, the suffix array interval [*b*, *e*[ contains all suffixes of *T* whose *k*-length prefix equals the leftmost *k*-mer of node $id$, i.e., $\omega [0,k[$. On line 4, the suffix array interval $[b', e'[$ is computed for the $(k+1)$-mer $c\omega [0,k[$ using basic functionality offered by the bidirectional FM-index. If this interval is non-empty (i.e., $c\omega [0,k[$ occurs in *T*), the identifier of the predecessor node is determined using function findIDRight (see Algorithm 1). Otherwise, the return value of $-1$ indicates that no such predecessor node exists. This routine can be called for all characters $c \in \Sigma$ to identify all predecessor nodes. Analogously, function getSuccIDWithChar illustrates how to find the successor node identifier during forward matching. Both functions execute in $\textrm{O}(1)$ time.
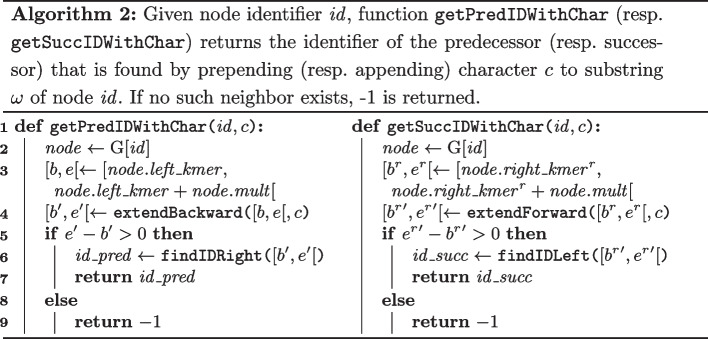

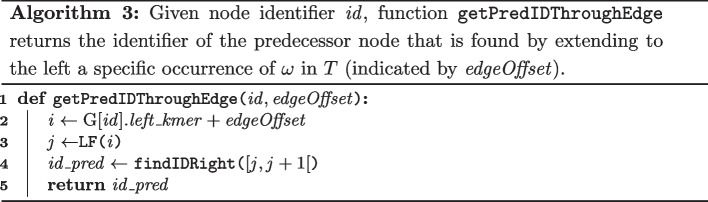


#### Jumping to a predecessor through a specific edge

Recall that the ccdBG $\textrm{G}(V,E)$ is a multigraph, i.e., there can be multiple edges between nodes *u* and *v*. With the exception of start and end nodes, each node has *mult* incoming and *mult* outgoing edges where *mult* corresponds to the number of times its substring $\omega$ occurs in *T*. Jumping to a predecessor through a specific edge is thus achieved by extending *a specific occurrence* of $\omega$ in *T* back to the predecessor node. All occurrences of $\omega$ in *T* of a node are represented in the interval $\textrm{SA}[ left\_kmer , left\_kmer + mult [$. A *specific* occurrence of $\omega$ in *T* is indicated by a relative offset $edgeOffset \in [0, mult[$ in this interval.

Algorithm 3 shows how the predecessor is found in this scenario. On line 2, value *i* is the $\textrm{SA}$ index such that the specific occurrence of $\omega$ starts at $T[\textrm{SA}[i]]$. The LF operation provided by the (bidirectional) FM-index computes the $\textrm{SA}$ index *j* such that $\textrm{SA}[j] = \textrm{SA}[i]-1$. Suffix $T_{\textrm{SA}[j]}$ thus has $c\omega$ as a prefix and we know that the *k*-length prefix of that suffix is the rightmost *k*-mer of the predecessor node of interest. Its identifier is found using the $\texttt {findIDRight}$ function from Algorithm 1. Assuming constant-time rank support on bit vectors, algorithm 3 runs in $\textrm{O}(1)$ time.

In the context of this paper, only jumping to a predecessor through a specific edge is required. Therefore, we omit its bidirectional counterpart in this section.

### Visualization

Using the elementary graph operations discussed before, the visualization of subgraphs of the pan-genome graph is achieved as follows. Given a set of seed nodes ($seedNodes$) of interest and a user-defined neighborhood size ($maxDepth$), Algorithm 4 generates a list of all nodes *u* for which $\textrm{distance}(u,v) \le maxDepth$ for some node $v \in seedNodes$. Here, $\textrm{distance}(u,v)$ is defined as the number of edges on the shortest path between *u* and *v*, irrespective of the orientation of edges. The time complexity of Algorithm 4 is $\textrm{O}(V_s|\Sigma |)$, with $V_s$ the number of nodes in the subgraph. It relies on the functions described in Algorithm 2. Similarly, Algorithm 5 lists all edges that are part of the subgraph, using the functionality provided by Algorithm 3. The subgraph can be visualized in e.g. Cytoscape [52].
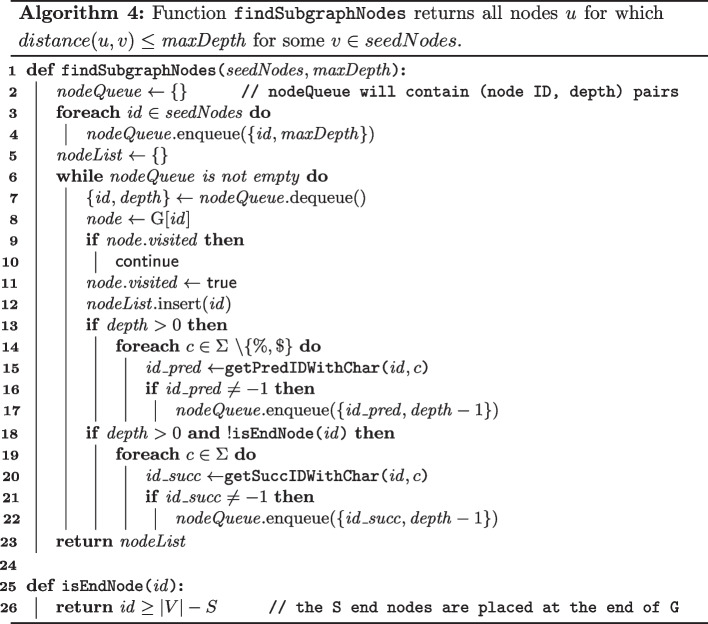

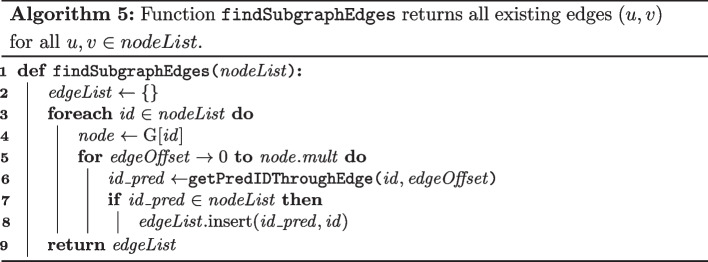


The visualization methodology proposed here differs from that of Dede and Ohlebusch [40] in that sense that their algorithms enable the visualization of only the *upstream* neighborhood of the region of interest, due to the fact that their use of the unidirectional FM-index supports only backward traversing of the graph. In contrast, our use of the bidirectional FM-index enables backward as well as forward traversing of the graph. Secondly, we separate the processes of matching patterns to the graph (see further) and visualizing subgraphs. Therefore, we can offer a very efficient pattern matching implementation, since we assume that users will mostly map great numbers of patterns to the graph, only few of which are interesting enough to be visualized. In Dede and Ohlebusch’s algorithms on the other hand, these processes are connected.

### Approximate pattern matching to the graph

In earlier work, Beller and Ohlebusch provided algorithms for exact pattern matching against the ccdBG. In this paper, we extend this functionality to also support *approximate* pattern matching, i.e., the identification of all approximate occurrences of a search pattern, allowing for substitutions, insertions or deletions. Formally, given a search pattern *P*, our implementation exhaustively identifies *all* occurrences *O* of search pattern *P* in *T* such that the edit distance $\textrm{ED}(O, P) \le K$. We support values $K = {0,1,2,3 \text { or } 4}$. Since the upper limit for the number of allowed errors is 4, our algorithms are most suited to identify occurrences of short, low-error (e.g., Illumina) reads or short seeds of long, higher-error (e.g., Pacific Biosciences, Oxford Nanopore Technologies) reads. The bidirectional FM-index and *search schemes* can be used to support lossless approximate pattern matching.

Once the occurrences *O* of a search pattern *P* have been identified, they can be located in the graph. Each approximate occurrence *O* of *P* is an exact substring of *T*. If the length of the occurrence *O* is at least the *k*-mer size, i.e., $|O| \ge k$, then *O* aligns to either a single node, or a unique sequence of connected nodes in the ccdBG. Otherwise, if $|O| < k$, then *O* can occur at multiple positions in the graph when *O* is a substring of multiple *k*-mers. For both cases, we provide algorithms in this section. Once a node path has been identified, a subgraph centered around this path can be extracted for visualization, using the functionality discussed earlier.

#### Search schemes

In lossless approximate pattern matching, all occurrences in search text *T*, within a certain Hamming or Levenshtein/edit distance of a query pattern *P*, are identified. In the context of this paper, the edit distance is used, allowing for up to *K* substitutions, insertions or deletions (collectively called errors). Using the FM-index, lossless approximate pattern matching is performed by spelling, character by character, candidate occurrences of *P* in *T*. Using a naive backtracking algorithm, an excessive number of unsuccessful branches near the dense root of the search tree will be explored, rendering backtracking computationally impractical even for modest values of *K* [44].

Kucherov et al. [42] proposed the concept of search schemes, which define how lossless approximate pattern matching should be conducted, such that the search space is strongly reduced. We adopt their notation. Pattern *P* is partitioned into *p* parts $P_i$, with $i \in \{0,...,p-1\}$. A search ${\mathcal {S}} = (\pi , L, U)$ is a triplet of arrays of size *p* where $\pi$ is a permutation over $\{0, ..., p-1\}$ that defines the order in which the parts of *P* are processed. It must satisfy the connectivity property in that sense that a partial match can only be extended, either to the left or to the right, in a contiguous manner. The arrays *L* and *U* define the lower and upper bound to the cumulative number of allowed errors after each part has been processed. The core idea is to only gradually increase the number of allowed errors when more parts of P are matched, significantly reducing the search space near the dense root of the search tree. To cover all possible error distributions over the length of a pattern, multiple searches are required that collectively form a search scheme. Search schemes require bidirectional matching functionality, i.e., a partial match *P* can be extended to $cP$ as well as $Pc$. This way, a pattern can be matched by starting with any part of *P* and then extending that partial match with adjacent parts, either to the left or to the right, in arbitrary order.

The simplest examples of search schemes are those based on the pigeonhole principle [41]. By partitioning search pattern *P* into $p = K+1$ parts, with *K* the maximum allowed number of errors, it immediately follows that for each occurrence of *P* in *T*, at least one part must be error-free. All occurrences are identified using $K+1$ searches ${\mathcal {S}}_i$. In search ${\mathcal {S}}_i$, exact matching of piece $P_i$ is performed first, and subsequently extended with the remaining pieces to the left and right, allowing up to *K* errors. For example, for $K = 2$ errors, the search scheme based on the pigeonhole principle is given by ${\mathcal {S}}_0 = (012, 000, 022)$, ${\mathcal {S}}_1 = (210, 000, 022)$ and ${\mathcal {S}}_2 = (102, 000, 022)$. Search ${\mathcal {S}}_2$ for example, starts with the exact matching of the middle piece $P_1$. Next, the match is extended to the left, and finally to the right, each allowing up to $K=2$ errors. This illustrates the need for a bidirectional index.

**Fig. 2:**
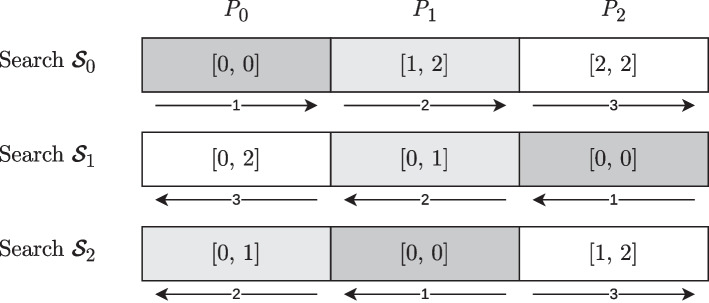
Search scheme by Kucherov et al. that allows up to 2 errors. The search scheme consists of three searches: ${\mathcal {S}}_0 = (012, 012, 022)$, ${\mathcal {S}}_1 = (210, 000, 012)$ and ${\mathcal {S}}_2 = (102, 001, 012)$. For each search, the processing order (from dark to light) and the lower and upper bounds for the cumulative number of errors after processing each part are indicated in the cells representing the parts. The arrows indicate the search direction (left-to-right or right-to-left). Search ${\mathcal {S}}_2$ for example, starts with the *exact* matching of the middle piece $P_{\pi [0]} = P_1$. Second, the match is extended to the left ($P_{\pi [1]} = P_0$), and third, to the right ($P_{\pi [2]} = P_2$). After processing part $P_0$ (and $P_1$), 0 or 1 errors should have been encountered. Similarly, after processing part $P_2$ *and* $P_0$ (and $P_1$), 1 or 2 errors should have been encountered. In summary, search ${\mathcal {S}}_2$ covers the following error distributions: [0, 0, 1], [0, 0, 2], [1, 0, 0] and [1, 0, 1]. It can be verified that every possible distribution of 2 errors among the three parts is covered by at least one of the three searches

Kucherov et al. proposed more efficient search schemes. Again, for the case of $K = 2$ errors, pattern *P* is partitioned into $K+1$ parts and the search procedure consists of three searches that are shown in Fig. 2. The key difference is that the search schemes by Kucherov et al. impose more stringent lower and upper bounds than those based on the pigeonhole principle, while still covering any distribution of errors over the different parts. In general, for larger values of *K*, search schemes can become quite complex to design and deviate significantly from the search schemes based on the pigeonhole principle. Kucherov et al. and Kianfar et al. [43] proposed search schemes for up to $K=4$ errors. The implementation of search schemes in Columba serves as a foundation for the work in this paper.

#### Identifying an occurrence in the graph

Search schemes allow to efficiently identify all occurrences *O* of search pattern *P* in *T* such that the edit distance $\textrm{ED}(O, P) \le K$, with *K* the maximum number of allowed substitutions and indels. Each occurrence *O* is represented by its suffix array intervals [*b*, *e*[ and $[b^r,e^r[$ and its length $l = |O|$ such that $O = T[\textrm{SA}[b], \textrm{SA}[b] + l[$. Analogously, the reverse occurrence $O^r$ is found as $O^r = T^r[\textrm{SA}^r[b^r], \textrm{SA}^r[b^r] + l[$. In other words, each approximate occurrence *O* of *P* is an exact substring of *T*.

In this section, we provide algorithms to identify the location in the graph that corresponds to *O*. If $|O| \ge k$, with *k* the *k*-mer size, *O* has a unique location in the ccdBG that can be represented by a sequence of connected nodes, along with a starting position in the first node. We consider the case $|O| < k$ later and assume for now that $|O| \ge k$.

The process involves two steps. First, we determine the node identifier for the leftmost *k*-mer of *O*. Next, we identify the node identifiers for the remaining part of *O*.

- *Step 1*: Determining the First Node Identifier

In general terms, identifying the node that contains the *k*-mer involves shifting, character by character, a *k*-length window through the node until a *k*-mer is found that can be used to identify the node. Until now, only the extreme (i.e., left- or rightmost) *k*-mers of a node could be used to obtain the node identifier (cf. Algorithm 1). However, the required number of shift operations can grow very large for long nodes and can even be $\textrm{O}(n)$ (with $n = |T|$) for large values of *k*. Therefore, we adapt bit vector $\textrm{B}$ and $\textrm{IDmap}$ such that determining the node identifier for an arbitrary *k*-mer can be achieved in constant time. Specifically, $\textrm{B}[i] = 1$ if the following two conditions apply:

1. *k*-mer $T[\textrm{SA}[i], \textrm{SA}[i]+k[$ has offset $(j \cdot s_{ cp })$ (for $j = 0, 1, 2, \ldots )$ in a node or is the rightmost *k*-mer of a node.
2. Suffix $T_{\textrm{SA}[i]}$ is the lexicographically largest suffix of *T* that has *k*-mer $T[\textrm{SA}[i], \textrm{SA}[i]+k[$ as a prefix.

In other words, besides the rightmost *k*-mer of each node, we also indicate every $s_{ cp }$th *k*-mer of a node. We refer to these extra *k*-mers as ‘checkpoint *k*-mers’. Their density is controlled by the user-defined checkpoint sparseness factor $s_{ cp }$. For the example in Fig. 1, and assuming a checkpoint sparseness factor of $s_{ cp } = 2$, *k*-mers “TAT” for node 1 and “GTT” and “TGG” for node 4 serve as checkpoint *k*-mers. Hence, three extra 1-bits in $\textrm{B}$ need to be set at indexes 13, 11 and 16 (see Table 2, in parentheses).

For each checkpoint *k*-mer, and hence, each additional 1-bit in bit vector $\textrm{B}$, a corresponding entry that points to the node identifier must be added to $\textrm{IDmap}$. Note that due to the checkpoint *k*-mers, the relationship between the 1-bits in $\textrm{B}$ and their corresponding nodes is now surjective, since multiple 1-bits are set in $\textrm{B}$ for nodes with $len >k$. We also add an extra row ($offset$) to $\textrm{IDmap}$ to identify the offset position of a *k*-mer within a node. As a consequence, $offset$ equals $len -k$ when the entry corresponds to a rightmost *k*-mer (the end nodes must again be extended cyclically), or $j \cdot s_ cp$ for each *j*th checkpoint *k*-mer. The extended $\textrm{IDmap}$ table corresponding to the example from Table 2 is illustrated in Table 6. Note that these modifications to bit vector *B* and $\textrm{IDmap}$ do not break the functionality of Algorithm 1.

**Table 6:** Extended $\textrm{IDmap}$ corresponding to Table 2, with $s_{ cp } = 2$

$id_{\textrm{B}}$	0	1	2	3	4	5	**6**	**7**	**8**	9
$id$	5	6	2	3	4	0	**4**	**1**	**4**	1
$offset$	2	2	0	0	3	0	**0**	**0**	**2**	2

First row: rank of the 1-bits in bit vector B; second row: node identifiers; third row: offset position of the *k*-mer with respect to the beginning of its node. Bold entries indicate checkpoint *k*-mers


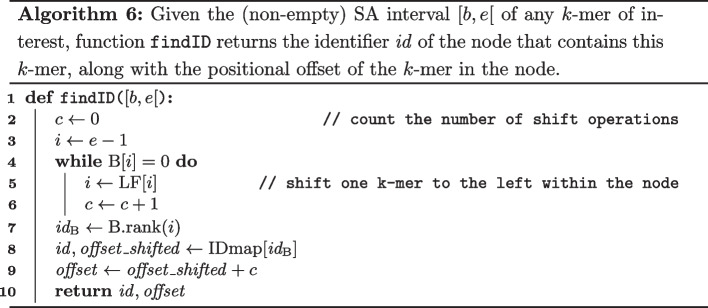


Given a *k*-mer, we use the basic functionality of the bidirectional FM-index to find its interval $\textrm{SA}[b,e[$ and $\textrm{SA}^r[b^r,e^r[$. We assume that the *k*-mer exists in *T*, i.e., these intervals are non-empty. Algorithm 6 shows how to retrieve the node identifier given the SA interval [*b*, *e*[ of the *k*-mer. On line 3, we consider the index *i* of the lexicographically largest suffix that has the *k*-mer of interest as a prefix. On lines 4 to 6, we consider the adjacent *k*-mers within the node, by advance to the left, character by character. More precisely, the LF operation returns the lexicographically largest index of the suffix prefixed by such an adjacent *k*-mer. This process continues until an index is encountered that is indicated by a 1-bit in bit vector B. In that case, the identifier and offset are retrieved on lines 7 to 8 in a similar manner as in Algorithm 1. By keeping track of the number of times the LF operation was used, the positional offset of the *k*-mer is easily computed on line 9.

For example, consider *k*-mer “TTG” with $\textrm{SA}$ interval [19, 20[ (see Table 2). Assume $s_{ cp }=2$. Because $\textrm{B}[19] = 0$, “TTG” is not the rightmost or a checkpoint *k*-mer of its node. Using the LF operation, we shift the *k*-length window one character to the left: $\textrm{LF}[19]$ yields index 11. Suffix $T_{\textrm{SA}[11]}$ is indeed prefixed by *k*-mer “GTT”. Because $\textrm{B}[11] = 1$ when $s_{ cp }=2$ (indeed, “GTT” is a checkpoint *k*-mer), we obtain $id = 4$ (see Table 6).

Note that the information on these checkpoint *k*-mers is only stored with respect to $\textrm{SA}$ (not $\textrm{SA}^r$): both the $\textrm{SA}$ range and the $\textrm{SA}^r$ range will always be available when we want to identify the node corresponding to an arbitrary *k*-mer (pattern matching to the bidirectional FM-index keeps track of both ranges in a synchronized manner).

In summary, at most $s_{ cp }-1$ LF operations are needed to find a *k*-mer that can be used to identify its node. Because the LF operation requires $\textrm{O}(1)$ time, the time complexity of Algorithm 6 is $O(s_{ cp })$. The user-defined parameter $s_{ cp }$ hence controls the time-space tradeoff.

- *Step 2*: Extending the Node Path

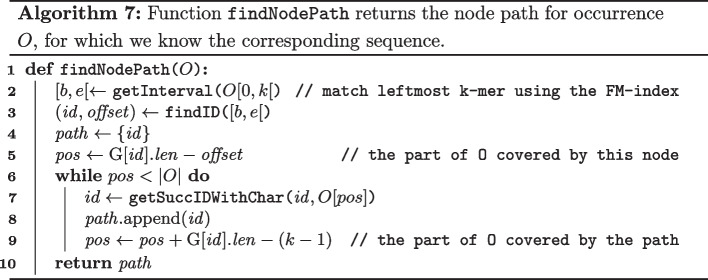


The sequence of nodes with which *O* aligns can now be easily identified as shown in Algorithm 7. In lines 2 to 5, the node identifier and start position of the leftmost *k*-mer *O*[0, *k*[ is found using the $\texttt {findID}$ function. Next, the nodes to which *O*[*k*, |*O*|[ aligns are identified in lines 6 to 9. Note that because we know that *O* is an exact substring of *T*, it is not necessary to match *O* character by character to the graph. Rather, for each visited node, one can immediately jump to the end of that node and use the getSuccIDWithChar function from Algorithm 2 to find the next node, etc.

The computation of the suffix array interval [*b*, *e*[ of *O*[0, *k*[ on line 2 of Algorithm 7 can be avoided. Recall that each occurrence *O* is generated character by character using search schemes. Therefore, it suffices to save the suffix array interval when the (partially generated) occurrence *O* reaches a length of *k*. Note that this interval does not necessarily correspond to the leftmost *k*-mer of *O*, as *O* can still be extended to the left and the right during the search scheme procedure. Nevertheless, it is easy to adapt Algorithm 7 such that one can start from any *k*-mer of *O* and then extend the path both to the left and right, using the bidirectional functionality offered by the data structure.

Finally, we consider the case $|O| < k$. This means that *O* could be found in multiple locations in the graph. In order to enumerate all locations, it suffices to enumerate, using the bidirectional FM-index, all possible *k*-length extensions of *O* that exist in *T*, and to identify the corresponding node for each such extension using the FindID function. This procedure can lead to redundant results, which can be filtered afterwards.

## Results and discussion

We implemented the algorithms of this paper in Nexus, an open-source tool written in standard C++14. The source code is available at https://github.com/biointec/nexus under AGPL-3.0 license.

### Data and hardware

We built pan-genomes of up to ten human genome builds also used in [40]: (i) five different assemblies of the human reference genome (UCSC Genome Browser assembly IDs: hg16, hg17, hg18, hg19, and hg38), (ii) the maternal and paternal haplotype of individual NA12878 (Utah female) of the 1000 Genomes Project [53], and (iii) three long read (PacBio) assemblies (GenBank assembly accession numbers: GCA_000001405.27, GCA_000002125.2 and GCA_000306695.2). All occurrences of ‘N’ were replaced by a randomly chosen nucleotide (‘A’, ‘C’, ‘G’ or ‘T’) to limit the alphabet size. The chromosomes within each build are concatenated into one string.

For benchmarking, we consider 100 000 Illumina HiSeq 2000 reads (101 bp) randomly sampled from a larger whole genome sequencing dataset (accession number ERR194147). All benchmark experiments were run on a Red Hat Enterprise Linux 8 system, using a single core of two 18-core Intel^®^ Xeon^®^ Gold 6240 CPUs running at a base clock frequency of 2.60 GHz with 738 GiB of RAM. Reported runtimes include the time for the approximate pattern matching procedure, but exclude the time to read the FM-index and graph data structures from disk.

We also conduct a case study on a pan-genome of 341 *M. tuberculosis* strains. Analogous to what was done in [54], we selected one reference strain of H37Rv (GenBank accession number CP003248.2), the assemblies of three historical isolates collected from KwaZulu-Natal [55, 56] (KZN4207, accession GCA_000669655.1; KZN1435, accession GCA_000669675.1; KZN605, accession GCA_000669635.1) and the assemblies of 337 clinical isolates, also collected from KwaZulu-Natal [54] (subset of BioProjects PRJNA183624 and PRJNA235618).

### Memory usage

#### Storing and using the data structure

Recall that we build our implicit pan-genome graph representation directly on top of the bidirectional FM-index as implemented in Columba. This additional graph representation, along with navigation functionality, comes at only a limited supplementary memory cost. Table 7 details the memory usage of the components of the bidirectional implicit representation of the ccdBG for a pan-genome of 10 human genomes, with $s_{\textrm{SA}} = 16$, $s_{ cp } = 128$ and $k=25$. The suffix array sparseness factor $s_{\textrm{SA}}$ is inversely proportional with the number of suffix array entries that are stored. This pan-genome consists of 30 340 521 923 characters, 66 102 955 graph nodes and 4 166 716 509 graph edges (not explicitly stored). The complete representation comprises 95.46 GiB, or approximately 27.03 bits per character.

**Table 7:** Overview of the components of Nexus’ data structure, with their respective memory usage

Component	Memory usage per entry [bits]	Number of entries		Total for 10 human genomes (GiB)
		General	10 human genomes	
Bidirectional FM-index	19.75 + 64/$s_{\textrm{SA}}$	*n*	~ 30 billion bp	83.89
$\textrm{G}$ $\textrm{B}$ $\textrm{B}^r$ $\textrm{IDmap}$ $\textrm{IDmap}^r$	192 1.25 1.25 64 32	|*V*| *n* *n* $|V| + |V|_ {cp}$ |*V*|	~ 66 million nodes ~ 30 billion bp ~ 30 billion bp ~ 137 million *k*-mers ~ 66 million nodes	1.48 4.42 4.42 1.02 252.16 MiB
Subtotal graph elements	-	-	-	11.57
**Total**	-	-	-	**95.46**

For each component, we indicate its number of entries and the number of bits per entry. The number of entries and memory usage of each component is illustrated for the pan-genome of 10 human genomes ($s_{\textrm{SA}} = 16$, $s_{ cp } = 128$, $k=25$)

The five components corresponding to the representation of the graph are stored as follows. Node vector $\textrm{G}$ stores attributes $len$ (32 bits), $mult$ (32 bits), $left\_kmer$ (64 bits), and $right\_kmer^r$ (64 bits) for each node. Bit vectors $\textrm{B}$ and $\textrm{B}^r$ support constant-time rank operations using the rank9 algorithm [51], i.e., 1.25 bits per character (25% overhead). Mapping $\textrm{IDmap}$ stores the node identifier (32 bits) and offset (32 bits) for all |*V*| rightmost *k*-mers and all $|V|_{cp}$ checkpoint *k*-mers. In the example pan-genome of 10 human genomes ($s_{ cp } = 128$ and $k=25$), 70 927 010 checkpoint *k*-mers are stored. Finally, $\textrm{IDmap}^r$ stores node identifiers (32 bits) for only the |*V*| leftmost *k*-mers. Note that unlike the bidirectional FM-index, these five components depend on the value of *k*.

In conclusion, the memory usage of the components corresponding to this pan-genome graph comprises less than 15% of the underlying bidirectional FM-index. This overhead is limited given the functionality that is provided to navigate and visualize the pan-genome graph. By building upon the underlying (bidirectional) FM-index in a complementary and modular way, future developments on index structures can likely be incorporated easily. The application of search schemes to the graph demonstrates this principle. However, the drawback of the bidirectional FM-index is that its space usage increases linearly with the pan-genome’s sequence content, limiting our current data structure to a few dozen human genomes. To address this, we plan to investigate the bidirectional *r*-index [57] as an alternative. The bidirectional *r*-index offers the same functionality as the bidirectional FM-index, but with sublinear index growth (i.e., proportional to the amount of *new* variation introduced by additional genomes incorporated into the pan-genome).

*State of the Art* Table 8 compares the memory usage of Nexus with that of other linear or graph pan-genome representations that can serve as a reference during read alignment. Both deBGA and Pufferfish represent the pan-genome as a (cc)dBG and use a *k*-mer hash table based data structure to index that (cc)dBG and label the unitigs with their corresponding occurrences in the input genomes. The memory usage of the index for deBGA and Nexus is quite similar, while Pufferfish is about 35% more space-efficient. However, note that unlike deBGA and Pufferfish, Nexus also provides other functionalities (such as visualization) next to read alignment. In contrast to a *k*-mer hash table, both the A4 algorithm by Beller and Ohlebusch and Nexus are based on a full-text index of the concatenation of all input genomes. Algorithm A4 builds its index based on the unidirectional FM-index, whereas Nexus utilizes the bidirectional FM-index, clarifying the increase in memory usage. Note that Nexus’ memory use can be reduced using parameter $s_{\textrm{SA}}$, see Additional file 1: Fig. S1. The indexes of BWA and Bowtie 2 are also based on the FM-index of the concatenation of all genomes in the pan-genome. In this regard, these linear indexes are conceptually highly similar to the underlying data structure of the graphs in A4 and Nexus. This similarity is also reflected in their reported memory usage. Finally, the Giraffe index comprises 2 to 5 times more memory than any other index discussed here.

**Table 8:** Comparison of Nexus ($s_{\textrm{SA}} = 16$) with other indexes that support read alignment either to a linear index (BWA, Bowtie 2), or to a graph which is used as a reference (Giraffe, deBGA, Pufferfish, A4)

Tool	Pan-genome representation	Memory usage data structure (GiB)	Construction time	Construction peak RAM usage (GiB)
deBGA	dBG	90.50	10 hours and 9 minutes	312.13
Pufferfish	ccdBG	62.29	17 hours and 27 minutes	139.24
A4	ccdBG	36.91	7 hours and 22 minutes	38.77
BWA	Linear concatenation	49.45	13 hours and 47 minutes	42.40
Bowtie 2	Linear concatenation	54.14	26 hours	246.88
Giraffe	Variation graph	180.04	21 hours and 3 minutes	638.93
Nexus	ccdBG	95.46	15 hours and 44 minutes	269.32

We report index memory usage, and index construction time and peak RAM usage for a pan-genome of 10 human genomes

#### Building the data structure

For the building process of the bidirectional implicit representation of the ccdBG, we prioritize limiting RAM usage over optimizing performance, as we believe the RAM usage to be the main bottleneck when building such large-scale graphs. The CPU and RAM usage of the building process depends on many factors:

- The more (diverse) input data, the more CPU time and RAM is needed.
- The smaller the suffix array sparseness factor $s_{\textrm{SA}}$, the less CPU time and the more RAM is needed.
- The lower parameter *k*, the more CPU time is needed.

The checkpoint sparseness factor $s_{ cp }$ has a relatively small impact on the graph construction process.

We built the data structure for a pan-genome of 10 human genomes with $s_{\textrm{SA}} = 16$, $s_{ cp } = 128$ and $k=25$. The complete process took 15 hours and 44 minutes, of which 41% was required for building the underlying bidirectional FM-index and the remaining time was used for constructing the implicit graph representation. Most of the former time period is spent building the regular and reverse suffix arrays. Most of the latter duration is used for building the longest common prefix (LCP) array (which is necessary to build the graph representation). The peak RAM usage is 269.32 GiB, which is reached during suffix array construction (the complete suffix array must be built before it can be stored in sparse form).

*State of the Art* Table 8 reports the CPU time and RAM required to build the index for Nexus and the other tools we compare with. We observe that the results for Nexus are in the same ballpark as those for deBGA, Pufferfish and Bowtie 2. A4 leverages a semi-external building process in order to limit the peak RAM usage, and it appears that this algorithm is also more efficient in terms of CPU usage. Also the BWA indexing process is more efficient than Nexus in terms of RAM usage. Building the variation graph using Giraffe was computationally more intensive than any of the other indexing processes, mainly in terms of RAM usage.

### Approximate pattern matching performance

#### Breakdown of Nexus’ performance

Due to the underlying bidirectional FM-index, Nexus provides a very efficient implementation of lossless approximate pattern matching against the ccdBG. That is, *every* occurrence that matches the pattern of interest within a specified maximum edit distance is reported, along with its corresponding positions in the sequences of the pan-genome. In Table 9, we analyze the runtimes for performing exact pattern matching using A4 and Nexus ($K=0$), and approximate pattern matching using Nexus ($K=\{1,2,3,4\}$). Note that A4 does not provide the option to match patterns approximately.

**Table 9:** Performance of A4 and Nexus for pattern matching against the graph for different maximum allowed edit distances: $K=\{0,1,2,3,4\}$

Maximum edit distance	A4 performance [reads/s]	Nexus performance [reads/s]		
		APM runtime [s]	Node path finding runtime [s]	Post-processing runtime [s]
*K* = 0	2 793 ± 278		13 324 ± 880	
		2.60 (34%)	2.03 (27%)	2.93 (39%)
*K* = 1	Not supported		3 274 ± 128	
		11.31 (37%)	3.80 (12%)	15.52 (51%)
*K* = 2	Not supported		1 145 ± 31	
		32.97 (38%)	9.36 (11%)	45.12 (51%)
*K* = 3	Not supported		472 ± 13	
		87.89 (41%)	23.17 (11%)	101.08 (48%)
*K* = 4	Not supported		199 ± 4	
		253.23 (51%)	52.27 (10%)	196.15 (39%)

We aligned 100 000 Illumina reads (length 101 bp) and their reverse complement to the pan-genome graph of 10 human genomes ($k=25$, $s_{ cp } = 128$ and $s_{\textrm{SA}} = 16$ – last two parameters only apply to Nexus). Runs were repeated 10 times. Performance (expressed in reads per second) is reported along with the 95% confidence intervals. Additionally, for Nexus, we provide a breakdown of the total runtime into the runtime for approximate pattern matching using the underlying FM-index, identifying the corresponding node path in the graph, and post-processing (identifying and filtering text occurrences)

As can be observed in Table 9, Nexus performs pattern matching 4.8 times faster than A4. Moreover, whereas A4 reports only the node path in the graph for each occurrence, Nexus also reports the position(s) with respect to the original reference text. For Nexus, Table 9 provides a breakdown of total runtime as follows: approximate pattern matching against the underlying FM-index (i.e., finding the SA interval(s)), finding the node paths corresponding to each occurrence found in the former procedure, and performing post-processing (i.e., finding all occurrences in the original reference text using suffix array accesses, extracting the corresponding pan-genome sequence identifier, and filtering these text occurrences). Note that a single search pattern can have multiple occurrences in the graph (in case of approximate pattern matching, i.e., $K > 0$) and that each individual occurrence in the graph can have multiple underlying text occurrences (in case it is repeated within or between strains). From this breakdown, we conclude that the fraction of time spent on finding node paths is limited. For approximate pattern matching to the graph, extracting the node path corresponding to the occurrences only requires about 11% of the total runtime. In contrast, the post-processing step (which is only present in sequence-to-graph aligners that report coordinates with respect to the underlying reference sequences) requires a substantial amount of time (40 to 50%).

#### Alignment sensitivity analysis

In Table 10, we compare the alignment results of Nexus (for different values of *K*) with other tools that support read alignment to the pan-genome as a reference in some form. Note that unlike the other aligners, Nexus (and A4) is currently more of a proof-of-concept implementation as it lacks support for certain features such as SAM output. Aligner deBGA is left out of the comparison for a few reasons. The authors state that deBGA is mainly focused on paired-end read alignment and may therefore not provide strong support for single-end read alignment. With a small alteration to deBGA’s code we got the single-end functionality running, but for reads with a large number of text occurrences, a segmentation fault occurs.

**Table 10:** Comparison of the alignment results of Nexus with other tools that support read alignment to the pan-genome in some form

Tool	Avg. nr. of text occurrences/read	Avg. nr. of graph occurrences/read	Fraction of aligned reads	Performance		Peak RAM usage [GiB]
				[reads/s]	[text occs/s]	
PuffAligner	11.79	Not reported	98.17%	7 216	85 724	62.44
A4	Not supported	0.90	89.39%	2 793	Not supp.	**38**.**57**
BWA-MEM	20.37/19.57	Not applicable	**99**.**95**/99.60%	1 430	29 113/27 975	49.80
Bowtie 2	9.92	Not applicable	99.56%	2 591	25 705	40.08
Giraffe	7.80/7.68	Not reported	98.36/96.95%	18	143/141	284.53
Nexus, *K* = 0	28.38	0.90	89.39%	**13 324**	**378 198**	90.71
Nexus, *K* = 1	94.01	2.14	95.11%	3 274	307 831	90.71
Nexus, *K* = 2	228.05	7.88	96.69%	1 145	261 124	98.08
Nexus, *K* = 3	450.76	23.17	97.47%	472	212 799	99.08
Nexus, *K* = 4	**782**.**70**	**46**.**56**	97.98%	199	156 133	100.59

For Nexus, we perform approximate pattern matching with different maximum allowed edit distances: $K=\{0,1,2,3,4\}$. We ran 10 experiments for aligning 100 000 Illumina reads of length 101 bp as well as their reverse complement to the pan-genome of 10 human genomes. The pan-genome dBGs are built for $k=25$, Nexus’ additional parameters are $s_{ cp } = 128$ and $s_{\textrm{SA}} = 16$. In terms of alignment output, we report the average number of reported text occurrences (i.e., in the form of a coordinate with respect to one of the input sequences) per read, the average number of graph occurrences (i.e., in the form of a node path) per read, and the fraction of reads that has at least one occurrence. In terms of performance, we report the average number of reads aligned per second, the average number of text occurrences reported per second, and the peak RAM usage of the alignment process. For BWA-MEM and Giraffe, two sets of text occurrences are reported: one with clipped alignments included and one with clipped alignments filtered out. For Bowtie 2 and Giraffe, the maximum number of alignments per read is capped at 10

The best result in each column is indicated in bold

The first thing that stands out, is the difference in number of reported text occurrences between Nexus and any of the other tools, which highlights the difference in sensitivity between lossy and lossless aligners. The average number of text occurrences per read varies around 10 for all of the lossy aligners, with a maximum of 20.37 for BWA-MEM. This is unsurprising since the pan-genome consists of 10 genomes, and we expect some reads to appear at multiple positions due to duplications. From Nexus’ results however, we learn that these tools miss quite some alignments: even at $K=0$ (i.e., exact alignment), Nexus identifies an average number of 28.38 text occurrences per read. This is because some reads have an extremely high number of text occurrences in the pan-genome (up to 37 872 exact text occurrences). For such reads, only a limited subset of text occurrences (or none at all) is reported by the other tools. This trend continues for a higher number of allowed errors: the number of text occurrences increases exponentially, due to the highly abundant reads (up to 227 347 text occurrences for some reads at edit distance 4).

The average number of graph occurrences (i.e., node paths) per read shows a similar increasing trend, but not as pronounced. This was to be expected, as one graph occurrence can correspond to many text occurrences. A4 and Nexus (at $K=0$) produce identical results. None of the other tools report graph occurrences, which emphasizes a core difference with our approach: next to the text coordinates, Nexus reports the corresponding node paths that were identified in the graph, which can be used for visualization and downstream analysis.

From the fraction of aligned reads, we learn that the state-of-the-art aligners are able to align slightly more reads than Nexus. This is a consequence of the hard limit of edit distance 4, which is imposed by Nexus as of now, while the other aligners are able to detect occurrences that have 5 (or more) errors. To address this limitation, Nexus can easily be extended to support search schemes for edit distance 5 (or higher). Moreover, we intend to develop an implementation for gapped alignment as well, to support the detection of longer indels.

*In-Depth Pairwise Sensitivity Comparisons* Upon performing an in-depth pairwise analysis of the aligned reads by Nexus ($K=4$) versus graph aligners PuffAligner or Giraffe (Additional file 1: Fig. S2), two distinct subsets of reads were each time identified, which could only be aligned by one of the tools. These subsets characterize the different search spaces of the two aligners. In both cases, the reads that are exclusively aligned by Nexus often correspond to a high number of text occurrences (i.e., multimapping reads). The reads exclusively aligned by PuffAligner or Giraffe tend to correspond to 10 or 8 text occurrences, respectively. These occurrences contain over 4 errors and are therefore missed by Nexus. Giraffe has an unexpected median of 8 text occurrences per read. Moreover, the distribution of Giraffe’s text occurrences across the 10 genomes in the pan-genome is uniform (Additional file 1: Fig. S3). These observations indicate that Giraffe is unsuitable for aligning reads to pan-genomes consisting of multiple *complete* genomes.

**Fig. 3:**
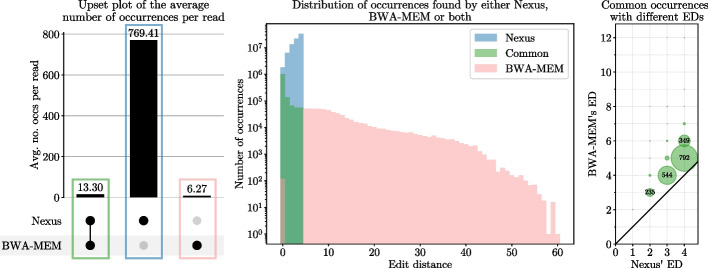
Analysis of the read alignment results of BWA-MEM (without clipped alignments) and Nexus ($K=4$) for mapping 100 000 Illumina reads of length 101 bp as well as their reverse complement to the pan-genome graph of 10 human genomes. Left: upset plot that shows the average number of occurrences per read reported by both tools, or exclusively by one tool. Middle: distribution of the reported number of occurrences (on logarithmic scale) in function of the corresponding edit distance. We distinguish occurrences reported by both tools (plotted in function of the edit distance reported by Nexus), and those reported uniquely by only one of the tools. Right: scatter plot visualizing the common occurrences where the edit distance reported by Nexus does not match that of BWA-MEM. In total, 2 258 such occurrences are observed, 9 of which are reported at an edit distance of 23 and 3 by BWA-MEM and Nexus, respectively (not shown in the scatter plot). Additional file 1: Table S2 lists the same information

A pairwise comparison between Nexus and linear aligners BWA-MEM and Bowtie 2 reveals that, in this case, there are (virtually) no reads exclusively aligned by Nexus (not shown). Upon further investigation however, we observe that although these linear aligners excel at identifying at least one occurrence for nearly all reads, they are more insensitive to detecting all (almost) equally good alternative alignments. Figure 3 shows a detailed comparison of the occurrences reported by Nexus and BWA-MEM. The left panel demonstrates that Nexus identifies 40 times more occurrences than BWA-MEM, 98.30% of which are reported exclusively by Nexus. On the other hand, 32.05% of BWA-MEM’s alignments are not found by Nexus. The middle panel of Fig. 3 shows that, apart from 118 exceptions, the reads exclusively aligned by BWA-MEM correspond to an edit distance larger than 4, which falls outside Nexus’ current limitations. These 118 exceptions are in fact also reported by Nexus, but at a slightly different coordinate in the reference genome. Furthermore, Nexus is guaranteed to report each alignment with its minimal edit distance (right panel of Fig. 3). Additional file 1: Figure S4 illustrates how Nexus accurately reports the minimal edit distance for an example read. The same analysis conducted with Bowtie 2 yields similar conclusions (see Additional file 1: Fig. S5; Table S3). In summary, Nexus’ core strength is complete sensitivity within its defined limitations, whilst BWA-MEM for instance only finds 35.75% of all exact alignments and only 0.16% of all alignments within an edit distance of 4.

#### Alignment performance analysis

Table 10 also reports three performance metrics. In terms of CPU time usage, PuffAligner, BWA-MEM and Bowtie 2 are the most efficient fully functional aligners in our comparison. Based on the number of reads mapped per second, Nexus is almost two times faster than PuffAligner at $K=0$, and faster than BWA-MEM and Bowtie 2 even at $K=1$. For higher values of *K* on the other hand, Nexus appears to be slower. However, based on the number of reported text occurrences per second, Nexus is significantly faster than any other aligner in our comparison. In other words, Nexus’ perceived lower performance per read is primarily due to it reporting a larger number of occurrences compared to other tools. In cases where the focus is solely on obtaining the optimal alignment(s) instead of all possible alignments within a specific edit distance, we propose a multi-stratum design gradually increasing the value of *K* until the optimal alignment(s) for a read are found. As such, the same alignment fraction reported for $K=4$ can be reached at a much higher speed. The results for peak RAM memory usage are similar to what was reported in Table 8. In conclusion, despite Nexus detecting *all* occurrences within a specified edit distance, it achieves similar or even better performance levels compared to its competitors.

#### The effect of $s_{ cp }$ and *k* on Nexus’ memory usage and APM performance

**Fig. 4:**
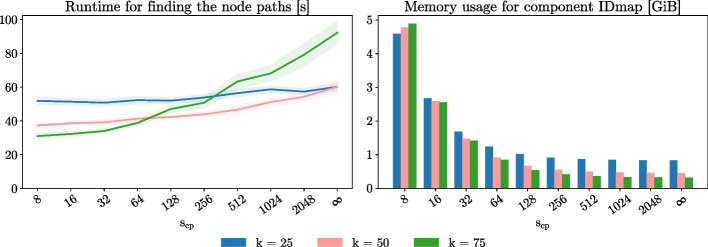
Left: average runtime over 10 runs for finding the node paths corresponding to the occurrences of 100 000 Illumina reads of length 101 bp and their reverse complement to the pan-genome graph of 10 human genomes, as a function of the checkpoint sparseness factor $s_{ cp }$ (8 to $\infty$). To find the occurrences, we performed approximate pattern matching with a maximum allowed number of errors of $K=4$, using the search scheme proposed by Kucherov et al. The pan-genome is built for $k=25$, $k=50$ and $k=75$, and $s_{\textrm{SA}} = 16$. The 95% confidence intervals for the runtime are also indicated. Right: total memory usage of the $\textrm{IDmap}$ component as a function of $s_{ cp }$

The use of checkpoint *k*-mers reduces the time complexity to identify the node in the graph that corresponds to an arbitrary *k*-mer to constant time at the cost of higher memory requirements. In Fig. 4, we analyze this time-space tradeoff by performing APM on a pan-genome of 10 human genomes for different values of $s_{ cp }$ and *k*. We also benchmarked without using checkpoint *k*-mers ($s_{ cp }=\infty$). We observe that for $k=50$ and $k=75$, decreasing $s_{ cp }$ results in faster node path extraction (Fig. 4, left). For $k=25$, we see that $s_{ cp }$ has only a limited effect on runtime. This is because, in that case, the median node length is short (28 characters), which means that even without intermediate checkpoint *k*-mers, only few LF-iterations are required to identify the node.

The total memory usage of component $\textrm{IDmap}$ is similar for all three values of *k* (Fig. 4, right). This is because where the average number of checkpoint *k*-mers per node is lower for low values of *k*, the higher number of nodes in the graph cancels out this effect (and vice versa). Note that the memory usage at its highest point ($k=75$, $s_{ cp }=8$) amounts to 4.89 GiB. This is a significant increase with respect to component $\textrm{IDmap}^r$ (83.90 MiB), but still only about 5% of the total data structure (Table 7). For a good balance between better APM performance and limited additional memory usage, we recommend a checkpoint sparseness factor around 128 (default). Finding the node paths for $k=75$ using this default value of $s_{ cp }=128$ for example, is twice as fast than without checkpoint *k*-mers with only 228 MiB additional memory usage.

### Case study on the bacterium *M. Tuberculosis*

In this case study, we demonstrate the potential of visualizing subgraphs and extracting information from the pan-genome graph topology. Specifically, we want to study antibiotic resistance in bacteria, as it remains a medically relevant topic for monitoring infectious diseases [54, 58–60]. Therefore, we built a pan-genome containing 340 *M. tuberculosis* strains from KwaZulu-Natal and one H37Rv reference strain, with $k=19$ (which was chosen after manual investigation), to visualize and investigate regions that are related to rifampicin resistance. Cohen et al. [54] listed 18 mutations from the RRDR region (the Rifampicin Resistance Determining Region, i.e., the 81 bp core region of gene *rpoB*), which is known to be related to rifampicin resistance [61]. From these mutations, we select the three that were reported to be observed in more than 50 strains of the dataset for closer investigation, as to limit the extent of this case study. Table 11 shows these three mutations, along with their coordinates with respect to the reference strain and the number of strains in the dataset that carry it.

**Table 11:** Overview of the mutations in the RRDR region in the *rpoB* gene of *M. tuberculosis* reported in [54], that are observed more than 50 times

Polymorphism identifier	Coordinate	Number of strains
D435G	761112	51
S450L	761157	88
L452P	761163	69

Coordinates are reported with respect to the H37Rv reference strain (zero-based indexing). We also show the number of strains in the 341-strain dataset that carry the mutations of interest

**Fig. 5:**
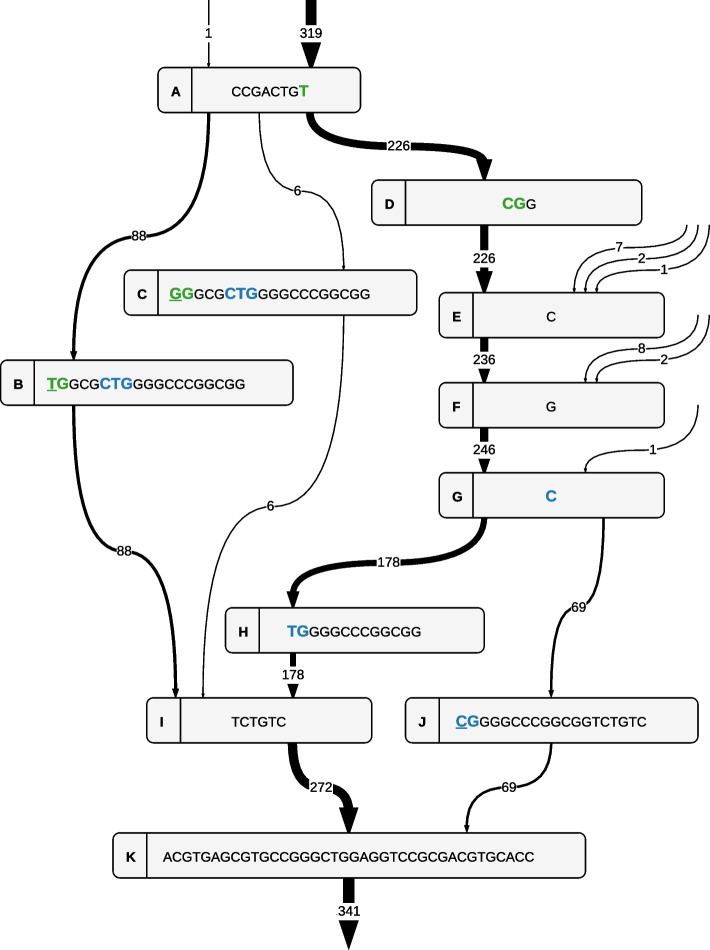
Visualization of a subgraph of the pan-genome ccdBG of 341 *M. tuberculosis* strains ($k=19$), corresponding to the end of the RRDR region of gene *rpoB*. The first $k-1$ overlapping characters have been omitted from each node and numerical node identifiers were replaced by characters A, B, etc., for clarity. The original subgraph is shown in Additional file 1: Fig. S6. Parallel edges are collapsed into a single edge, shown with its multiplicity. Edge thickness also reflects multiplicity. The reference H37Rv strain follows the path of the dominating edges (i.e., node path ADEFGHIK). Except for the lateral inflow of 21 strains on the right (due to other mutations in upstream regions), we observe that there are three alternative paths from node A to node K: through node B, C or J. These alternative paths are present due to mutations in codons 450 and 452 (“TCG” and “CTG” in the reference), which are shown in green and blue. Specifically, mutations S450L, S450W (not present in Table 11 as it is only observed in 6 strains), and L452P are underlined (codons “TTG”, “TGG”, and “CCG” in nodes B, C, and J, respectively)

Using the visualization algorithms discussed earlier, these mutations with their surroundings can be visualized. As the visualization of the complete RRDR region is too large to include in this paper, we show the subgraph that contains mutations S450L and L452P (i.e., the end of the RRDR region) in Fig. 5. Such visualizations are beneficial during hands-on research, as they allow the end-user to manually investigate the regions of interest in depth. In this case, we can indeed confirm that the mutations observed in [54] are present in our dataset.

#### Compensatory mutations

Cohen et al. report, “While accumulation of drug-resistance mutations can confer a fitness cost to bacteria, subsequent development of compensatory mutations can ameliorate these costs by restoring certain affected physiological functions while maintaining drug resistance” [54]. For rifampicin resistance, putative compensatory mutations in genes *rpoA*, *rpoC* and the non-RRDR regions of gene *rpoB* have already been discussed in previous literature [62–64]. Cohen et al. analyzed these reported compensatory mutations and investigated the pan-genome for new ones. In total, they report 49 putative rifampicin compensatory mutations that meet their requirements (i.e., evolved after or concurrent to genotypic rifampicin resistance), 26 of which were newly identified. In this paper, we only consider putative compensatory mutations that are co-mutated with one of the three mutations in Table 11, and that occur at least twice. These limitations leave us with 15 putative compensatory mutations, which are reported in the second column of Table 12.

**Table 12:** Overview of the *candidate* putative compensatory mutations in *rpoA*, *rpoC* and the non-RRDR regions *rpoB*

Coordinate	Mutation name	Node identifier	RRDR mutation	Nr. of strains
761246	I480V	97472	S450L	2
761549	V581M	55246	S450L	4
762199		111083	S450L	2
762279	R824L	105067	S450L	2
762287	R827C	109947	S450L	8
762733	Q975H	108299	S450L	5
762891	H1028R	82684	L452P	6
763125	I1106T	61206	L452P, D435G	50
764365	G332S	50267	S450L	2
764819	V483G	96203	S450L	2
765621	E750D		S450L	2
766487	V1039A	56977	S450L	9
766489	P1040S	105810	S450L	18
766490	P1040R	92909	S450L	3
767125	V1252L	115316	S450L	5
3878138	V183G		S450L	3

We report the coordinate with respect to the reference H37Rv strain; the name of the mutation (if it is reported in [54]); the node identifier in the graph ($k=19$) of the candidate (if it is reported by our pipeline); the co-mutated RRDR polymorphism, i.e., the mutation from Table 11 which is also carried by all strains that pass through this candidate; and the number of strains in the dataset that correspond to this candidate

In this paper, we set up an independent search for putative compensatory mutations by leveraging the functionality of finding the neighboring nodes of a certain node path. Specifically, we find the neighboring nodes in the graph for genes *rpoA*, *rpoC* and the non-RRDR regions of gene *rpoB* in the H37Rv reference strain, using the visualization algorithms. If possible, we assign a coordinate to the neighboring nodes, by jumping back to predecessor nodes (Algorithm 3) until a node is encountered that can be unambiguously positioned with respect to reference H37Rv (details are omitted). We then consider a neighboring node to contain a *candidate* putative compensatory variation if the following conditions are met:

1. A coordinate was found within a limited number of steps back in the graph.
2. The neighboring node contains *only* strains that carry one of the three mutations from Table 11. All strains must carry the *same* RRDR mutation.
3. The neighboring node must have a multiplicity of at least two. In other words, the *candidate* putative compensatory variation must appear at least twice.

Applying this workflow to our pan-genome, results in 14 *candidate* putative compensatory mutation nodes, for which their coordinate (with respect to reference H37Rv), node identifier, corresponding RRDR mutation and multiplicity is shown in Table 12. Note that most candidate compensatory genes correspond to the S450L RRDR mutation, as was also observed in [54]. We compare our results with the 15 putative compensatory mutations reported in [54] we selected previously. We can distinguish three categories in Table 12: 2 entries were only reported in [54], 1 entry was only reported by our pipeline, and 13 entries correspond to a matching polymorphism reported both in [54] and by our pipeline.

Two polymorphisms are missed by our pipeline due to the following reasons:

- E750D: the transition from glutamic acid to aspartic acid happens in two ways: from “GAG” to “GAC” (node 67460) and from “GAG” to “GAT” (node 108256). Hence, they are presented as two separate mutations with a multiplicity of one, which do not meet the third condition.
- V183G: this mutation can be found in node 61254, but one of the strains that passes through it is not genotypically rifampicin resistant (i.e., does not carry an RRDR mutation). Hence, this node does not meet the second condition.

In summary, our pipeline detects all putative compensatory mutations from [54] within the limits we imposed.

**Fig. 6:**
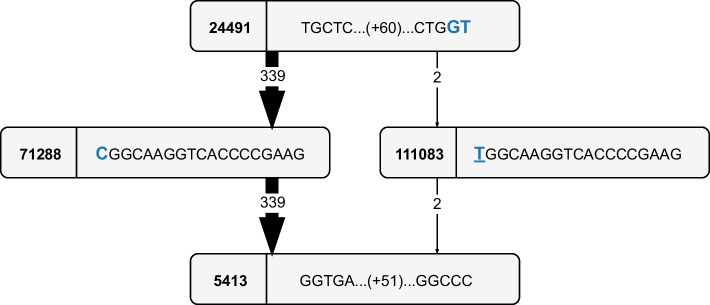
Visualization of the new *candidate* putative compensatory mutation at coordinate 762199 from Table 12, as a subgraph of the pan-genome ccdBG of the 341-strain *M. tuberculosis* dataset ($k=19$). The first $k-1$ overlapping characters have been omitted from each node for clarity. Parallel edges are collapsed into a single edge, shown with its multiplicity. Edge thickness also reflects multiplicity. The reference H37Rv strain follows the path of the dominating edges (i.e., node path 24491-71288-5413). Node 111083 is reported by our pipeline to be a *candidate* putative compensatory variation. The mutation of interest happens in codon 797, which is indicated in blue. Specifically, codon “GTC” is altered to codon “GTT”, which both translate to amino acid valine. Hence, this variation is a silent mutation

For the entry that is only reported by our pipeline, further research is required. First, we investigate the type of the variant: it could be a substitution (silent, missense or nonsense), or an insertion/deletion (possibly introducing frameshift). We do this manually, based on the visualization of the neighborhood of this variation (see Fig. 6) and the codon information of the reference genome on NCBI. As is detailed in Fig. 6, node 111083 contains a silent mutation, which is currently not known to have compensatory effects. If a missense mutation had been found, additional research would have been required (e.g., verifying the presence of this candidate in multiple, distinct phylogenetic clades or verifying that this candidate evolved after or concurrent to genotypic rifampicin resistance).

Note that the pipeline discussed in this section is more of an ad hoc solution to the problem of finding candidate compensatory mutations corresponding to mutations in the RRDR region of *rpoB*, rather than a general pipeline to be readily applied to other problems. Moreover, the choice of parameter *k* has a big impact on the results of this pipeline. Nevertheless, the results from [54] can be reproduced within the limitations we impose, without the need for variant calling. Hence, this application clearly shows the potential of exploiting the information that is embedded in the pan-genome ccdBG, using the graph operations we propose in this paper.

## Conclusions

In this paper we proposed Nexus, a memory-efficient representation of the colored compacted de Bruijn graph enabling subgraph visualization and lossless approximate pattern matching of reads to the graph, developed to store pan-genomes. This implicit graph representation is built on top of the bidirectional FM-index in a modular and complementary way, with a limited additional memory cost (around 15%). We demonstrated that it allows for easy integration of recent developments for the bidirectional FM-index, by applying search schemes to our pan-genome graph. Using search schemes, we provided a very efficient implementation of lossless approximate pattern matching of reads to the graph, showing similar performance to state-of-the-art lossy read(-to-graph) aligners. We showed that Nexus’ strength is to identify all possible occurrences corresponding to a read, even if they are highly abundant. We also established a use-case demonstrating the advantage of Nexus’ versatility, by combining both the approximate pattern matching and visualization functionalities to analyze antimicrobial resistance mutations and their possible compensatory mutations. Future work includes extending the implementation to a complete aligner (e.g., providing SAM output), integrating paired-end read alignment, building a multi-stratum search scheme design and extending the search schemes to allow for more than 4 errors.

### Supplementary Information

**Additional file 1**. Supplementary information on the bidirectional FM-index, supplementary results, and instructions for reproducing the results

## Data Availability

The datasets supporting the conclusions of this article are publicly available, and the ‘Data and Hardware’ section lists all corresponding dataset identifiers and references. The C++ source code of Nexus is available at https://github.com/biointec/nexus under the GNU AGPL v3.0 license.

## Abbreviations

AGPL: Affero general public license
AMR: Antimicrobial resistance
APM: Approximate pattern matching
BWA: Burrows-Wheeler aligner
BWT: Burrows-Wheeler transform
ccdBG: Colored compacted de Bruijn graph
cdBG: Compacted de Bruijn graph
CPU: Central processing unit
dBG: de Bruijn graph
DNA: Deoxyribonucleic acid
DP: Dynamic programming
ED: Edit distance
ID: Identifier
LCP: Longest common prefix
LF: Last-to-First (property)
MEM: Maximal exact match
NCBI: National center for biotechnology information
ONT: Oxford nanopore technologies
PacBio: Pacific biosciences
RAM: Random-access memory
RRDR: Rifampicin resistance determining region
SA: Suffix array
SNP: Single-nucleotide polymorphism
UCSC: University of California Santa Cruz
