# Supplementary material for: Pan-genome de Bruijn graph using the bidirectional FM-index
Source: BMC Bioinformatics. 2023 Oct 26;24:400. doi: 10.1186/s12859-023-05531-6 (PMC10605969; doi:10.1186/s12859-023-05531-6)
Supplement: Supplementary file 1 — Additional file 1. Supplementary information on the bidirectional FM-index, supplementary results, and instructions for reproducing the results [file 12859_2023_5531_MOESM1_ESM.pdf]

Pan-genome de Bruijn Graph using the Bidirectional FM-index: Supplementary Material

Lore Depuydt[1*], Luca Renders[1], Thomas Abeel[2,3] and Jan Fostier[1*]

*Correspondence:
Lore.Depuydt@UGent.be;
Jan.Fostier@UGent.be
[1]Department of Information Technology - IDLab, Ghent University - imec, Technologiepark 126, B-9052 Ghent (Zwijnaarde), Belgium
Full list of author information is available at the end of the article

1 Supplementary Methods

1.1 Bidirectional FM-index

In this section, we provide a brief overview of the bidirectional FM-index, which forms the base of our pan-genome graph representation.

The Burrows-Wheeler transform $\text{BWT}[0..n[$ of a text T of length n is defined as $\text{BWT}[i] = T[\text{SA}[i] - 1]$ if $\text{SA}[i] > 0$ and $\text{BWT}[i] = \$$ otherwise [1]. Here, SA denotes the suffix array, an array over integer values that indicate the starting positions of the suffixes of T in lexicographic order [2]. We build the suffix array using `divsufsort` (coded by Yuta Mori). There is an important relationship between the characters in the sorted permutation of T (called F) and the BWT of T (called L), namely the LF property. The LF property states that the ith occurrence of a particular character c in the BWT and the ith occurrence of c in F correspond to the same character in T. We thus need support for $\text{Occ}(c, i)$ queries on the BWT that return the number of occurrences of character c in the prefix $\text{BWT}[0..i[$. We realize this using $|\Sigma|$ bit vectors with constant-time rank support (`rank9` algorithm [3]). The LF property can then be computed in constant time as follows: $\text{LF}[i] = \text{C}(c) + \text{Occ}(c, i)$, with $c = \text{BWT}[i]$. Here, $\text{C}(c)$ denotes the number of characters in T strictly smaller than c. These values are pre-computed and stored in a small array of size $|\Sigma|$. Collectively, the BWT, SA, bit vectors and C array are referred to as the (unidirectional) FM-index [4]. Often, only the suffix array entries for every s_{SA}th suffix are stored, s_{SA} being the suffix array sparseness factor. This sparse representation of the suffix array requires an auxiliary bit vector with rank support to indicate the presence or absence of index positions and to compute their offsets within the sparse suffix array. Every entry of the suffix array can be computed in $\text{O}(s_{\text{SA}})$ time using the LF property. This is one of the various design choices through the which time-space tradeoff can be controlled.

Exact pattern matching using the FM-index is performed by matching character by character, from right to left. Let $[b, e[$ denote the interval over the suffix array for which the corresponding suffixes have P as a prefix. The suffix array interval $[b', e'[=$ `extendBackward`$([b, e[, c)$ whose suffixes have cP as a prefix can then be computed by $b' = \text{C}(c) + \text{Occ}(c, b)$ and $e' = \text{C}(c) + \text{Occ}(c, e)$. Because $\text{Occ}(c, i)$ queries can be performed in constant time, exact matching of a pattern P of size m takes $\text{O}(m)$ time. The size of the obtained interval $[b, e[$ denotes the number of occurrences of P in T. The positions of the occurrences in T can be obtained using the suffix array.

A bidirectional FM-index is obtained by also storing BWT^r, the Burrows-Wheeler transform of T^r, the reverse of T. By keeping track of both the range $[b, e[$ over BWT as well as the range $[b^r, e^r[$ over BWT^r in a synchronized manner, one can extend a pattern P to either cP (`extendBackward`) or Pc (`extendForward`) in $\text{O}(|\Sigma|)$ time [5].

Table S1 Overview of all components of our bidirectional FM-index, with their respective memory usage. For each component, we clarify the number of entries it contains, and the number of bits needed to store each entry. The number of entries and memory usage of each component is illustrated for the pan-genome of 10 human genomes ($s_{\mathrm{SA}} = 16$).

Component	Memory usage per entry [bits]	Number of entries		Total for 10 human genomes
		General	10 human genomes	
Compacted text	3	n	$\sim$ 30 billion bp	10.60 GiB
Counts array C	64	256	256 characters	2 KiB
BWT	3	n	$\sim$ 30 billion bp	10.60 GiB
PrefixOcc	6.25	n	$\sim$ 30 billion bp	22.08 GiB
PrefixOcc^r	6.25	n	$\sim$ 30 billion bp	22.08 GiB
Sparse SA	64	n/s_{SA}	$\sim$ 1.9 billion entries	14.13 GiB
SA bit vector	1.25	n	$\sim$ 30 billion bp	4.42 GiB
**Total**				83.89 GiB

By replacing the Occ data structure with a PrefixOcc data structure, this bound is improved to O(1) [6]. Note that both ranges always have the same width, which monotonically decreases when new characters are added to the occurrence (adding a character cannot lead to more occurrences in the reference text).

Table S1 details the components of the bidirectional FM-index that is used in our implementation. The compacted reference text uses 3 bits to distinguish 6 characters. Note that the original reference text T (8 bits per character), is necessary to build the representation. Once this process is finished, T can be removed from disk to spare memory. The counts array stores counts for each character in the extended ASCII alphabet, which is why 256 entries are stored. In practice, only 6 of these entries can have non-zero values ('A', 'C', 'G', 'T', '%' and '$'). BWT uses 3 bits per character, analogous to the compacted reference text. PrefixOcc, resp. PrefixOcc^r, stores the locations of characters 'A', 'C', 'G', 'T', '%' in BWT, resp. BWT^r, in 5 bit vectors (accounting for 5 bits per character). Additionally, each of these bit vectors must support constant-time rank operations, which leads to 0.25 bits overhead per character per bit vector (`rank9` algorithm [3]). This totals to 6.25 bits per character for tables PrefixOcc and PrefixOcc^r. Note that we only need PrefixOcc^r, not BWT^r itself. To guarantee O(s_{SA}) suffix array indexing, we store each s_{SA}th suffix in the sparse SA. To check whether a certain index in the SA corresponds to a s_{SA}th suffix or not, we need an additional bit vector indicating the stored entries. This bit vector also needs 0.25 additional bits per entry for constant-time rank support.

Finally, the longest common prefix array or the LCP array is also used for the construction of our pan-genome graph. The LCP array of string T, denoted by LCP, is an array of size $n+1$ such that LCP[0] $= -1$, LCP[n] $= -1$ and LCP[i] $=$ lcp($T_{\mathrm{SA}[i-1]}, T_{\mathrm{SA}[i]}$) for $0 < i < n$, where lcp(u, v) denotes the length of the longest common prefix between two strings u and v [7].

2 Supplementary Results

2.1 The Effect of the Suffix Array Sparseness Factor on Memory Usage and APM Performance

Adjusting the suffix array sparseness factor can lead to significantly less memory usage, as the complete suffix array is by far the largest component of the data

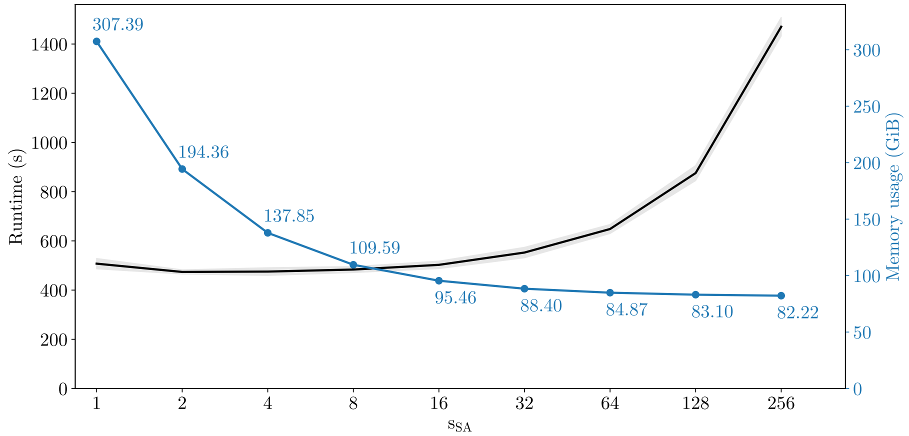

Figure S1 Black: average runtime over 10 experiments for mapping 100 000 Illumina reads of length 101 bp as well as their reverse complement to the pan-genome graph of 10 human genomes, as a function of the suffix array sparseness factor s_{SA} (1 to 256). We perform approximate pattern matching with a maximum allowed number of errors of $K = 4$, using the search scheme proposed by Kucherov et al.. The pan-genome is built for $k = 25$ and $s_{cp} = 128$. The 95% confidence interval for the runtime is also indicated. Blue: the memory usage of the complete representation of the ccdBG for the pan-genome of 10 human genomes (including the underlying bidirectional FM-index), as a function of the suffix array sparseness factor s_{SA}.

structure. Fig. S1 illustrates the time-space tradeoff that arises when s_{SA} is altered. It shows the runtime of the APM procedure for matching 100 000 Illumina reads to the pan-genome graph of 10 human genomes as well as its memory usage, as a function of s_{SA}.

For $s_{\mathrm{SA}} = 1$, the suffix array is stored at full capacity and the total memory usage is 307.39 GiB. When s_{SA} is increased, the memory usage of SA decreases as less of its entries are explicitly stored. For an infinitely large value of s_{SA}, the data structure comprises 81.33 GiB as the memory usage of SA approaches 0 GiB. The performance of the APM procedure decreases as s_{SA} increases, since the SA entries that are not explicitly stored must be recalculated on the fly. We see that the runtime increases exponentially with s_{SA}. Note however that for very small values of s_{SA} (1, 2, 4, 8), the APM performance is barely affected. In fact, the APM performance is better at $s_{\mathrm{SA}} = 2$ than at $s_{\mathrm{SA}} = 1$, presumably due to the high RAM requirements.

Determining the right value for s_{SA} is different in every scenario: large pan-genomes require a higher suffix array sparseness factor, whilst smaller datasets benefit from a more complete suffix array. Still, choosing $s_{\mathrm{SA}} = 16$ or $s_{\mathrm{SA}} = 32$ generally leads to a good balance.

2.2 Comparison of Nexus with Other Aligners

2.2.1 Comparison of Nexus with PuffAligner

Fig. S2 (a) shows the comparison of Nexus' alignment results with those of PuffAligner.

2.2.2 Comparison of Nexus with Giraffe

Fig. S2 (b) shows the comparison of Nexus' alignment results with those of Giraffe. Before conducting the analysis, all clipped alignments were discarded from Giraffe's

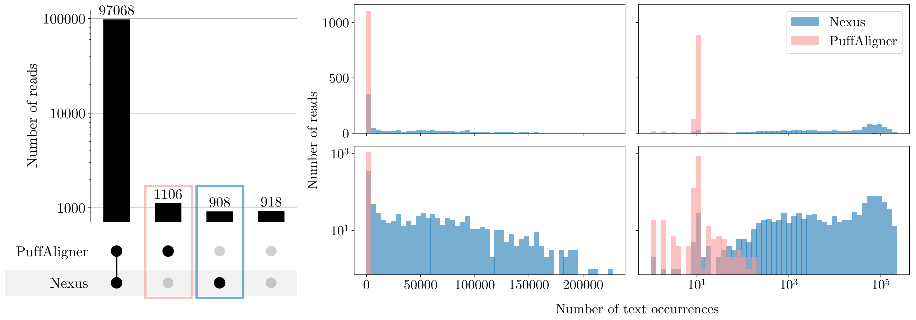

(a) Comparison of Nexus ($K = 4$) with PuffAligner

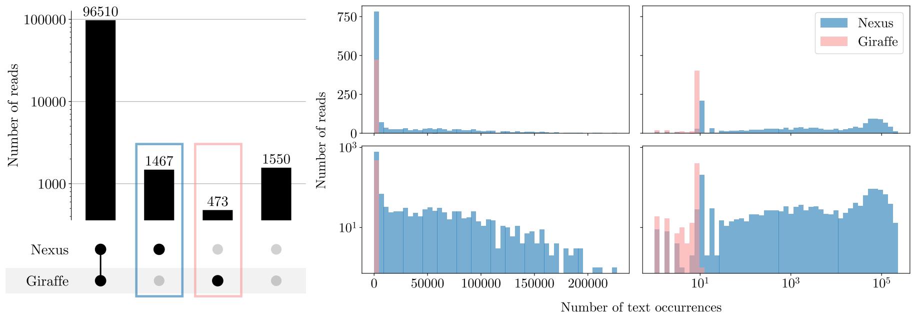

(b) Comparison of Nexus ($K = 4$) with Giraffe (without clipped alignments)

Figure S2 Analysis of the reported occurrences for different aligners for mapping 100 000 Illumina reads of length 101 bp as well as their reverse complement to the pan-genome graph of 10 human genomes. Left: upset plot that shows the number of reads that are aligned by both tools, by only one tool, or by none. Right: the distribution of reads aligned by only one of the two tools as a function of the corresponding number of reported text occurrences. The same histogram is shown in 4 forms to highlight different features of the distributions: on a linear and logarithmic axis for the number of text occurrences (left and right), and on a linear and logarithmic axis for the corresponding number of reads (top and bottom).

results because we are only interested in end-to-end alignments in the context of this paper. The number of alignments per read was cut off at 10 for Giraffe to reduce runtime, and because 10 is the expected number of alignments for most reads since 10 genomes are contained in the pan-genome. For this reason, no number of text occurrences higher than 10 is reported in the histograms. Fig. S3 shows the distribution of the number of text occurrences reported per genome by Giraffe.

2.2.3 Comparison of Nexus with BWA-MEM

Tab. S2 contains the same data as visualized in the right scatterplot of Fig. 3 in the paper. To illustrate that Nexus effectively reports a correct minimal edit distance, we investigate the occurrences corresponding to the first row of this table in Fig. S4.

2.2.4 Comparison of Nexus with Bowtie 2

Tab. S3 provides additional alignment results corresponding to Bowtie 2 for different values of the `-k` parameter (i.e., the maximum number of alignments that can be reported per read). The first row corresponds to the Bowtie 2 entry in Tab. 10 in the paper. Additionally, Fig. S5 provides an in-depth analysis of Nexus versus Bowtie 2

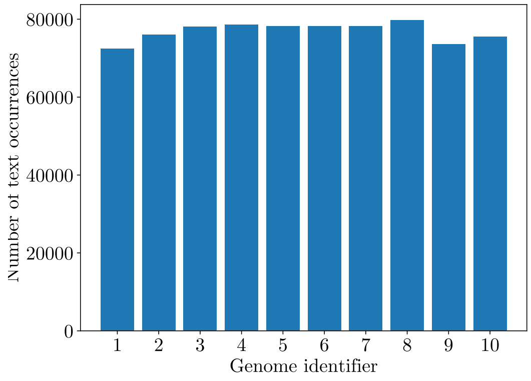

Figure S3 The number of text occurrences reported per genome by Giraffe, for matching 100 000 Illumina reads (length 101 bp) and their reverse complement to the pan-genome graph of 10 human genomes. It can be observed that on average, approximately 8 occurrences are reported per read, and these occurrences are distributed uniformly over the 10 included genomes.

...GGCGGAGGAGGA
...GGCGGAGGA - GA

(a) Nexus' alignment

...GGCGGAGGAG- -GA
...GGCGGAGGAGATGA

(b) BWA-MEM's alignment

Figure S4 The alignment that is reported at edit distance 1 by Nexus (a) and edit distance 2 by BWA-MEM (b). This alignment corresponds to all 3 occurrences of the first row of Tab. S2. Only the end of the alignment is shown because the beginning is an exact match for both tools. The top sequence each time represents the read, while the bottom sequence represents the aligned sequence from the reference genome.

for each of value of the `-k` parameter, analogous to the analysis conducted for BWA-MEM in the paper. In general, the same conclusions can be drawn for Bowtie 2 as for BWA-MEM. Note that for higher values of the `-k` parameter, Bowtie 2 detects more occurrences with an edit distance lower than 5 that are not found by Nexus. The explanation for this observation remains the same as for BWA-MEM.

2.3 Cytoscape Visualization

In Fig. 5 in the manuscript, we illustrate the visualization of a subgraph of the pan-genome of 341 *M. tuberculosis* strains in a curated form:

- the first $k-1$ overlapping characters were omitted from each node;
- node identifiers were replaced by a smaller set of character identifiers;
- irrelevant nodes were purged from the graph;
- the multiplicity of the edges was added to the figure explicitly.

In this section, we include the original subgraph as it is visualized by Cytoscape in Figure S6, which was created using the following command:

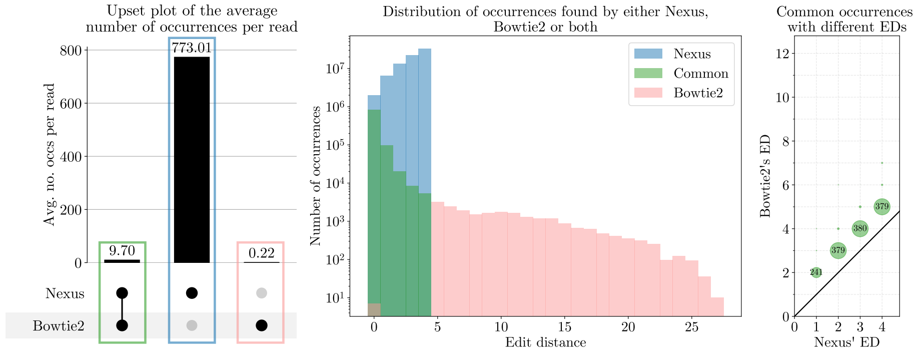

(a) Bowtie 2 `-k` 10

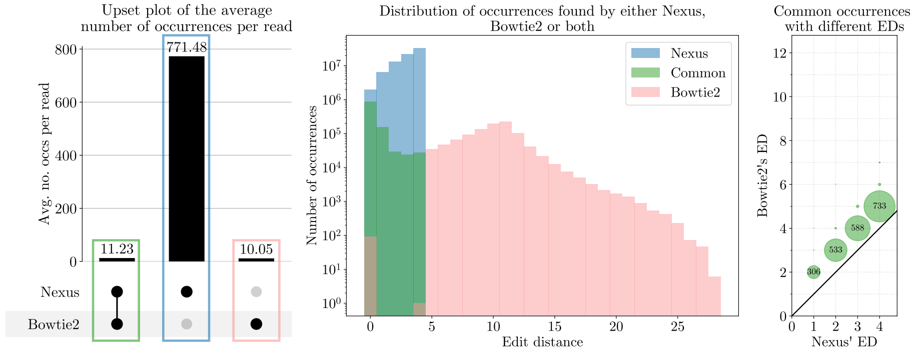

(b) Bowtie 2 `-k` 100

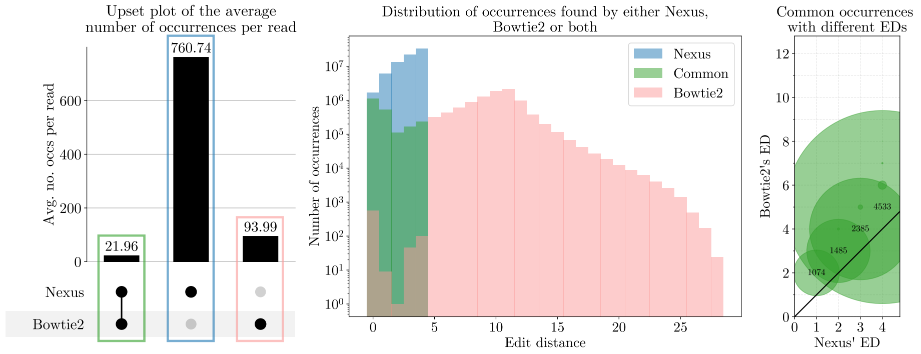

(c) Bowtie 2 `-k` 1000

Figure S5 Analysis of the read alignment results of Bowtie 2 (for different maximum numbers of alignments per read) and Nexus ($K = 4$) for mapping 100 000 Illumina reads of length 101 bp as well as their reverse complement to the pan-genome graph of 10 human genomes. Left: upset plot that shows the average number of occurrences per read reported by both tools, or exclusively by one tool. Middle: distribution of the reported number of occurrences (on logarithmic scale) in function of the corresponding edit distance. We distinguish occurrences reported by both tools (plotted in function of the edit distance reported by Nexus), and those reported uniquely by only one of the tools. Right: scatter plot visualizing the common occurrences where the edit distance reported by Nexus does not match that of Bowtie 2.

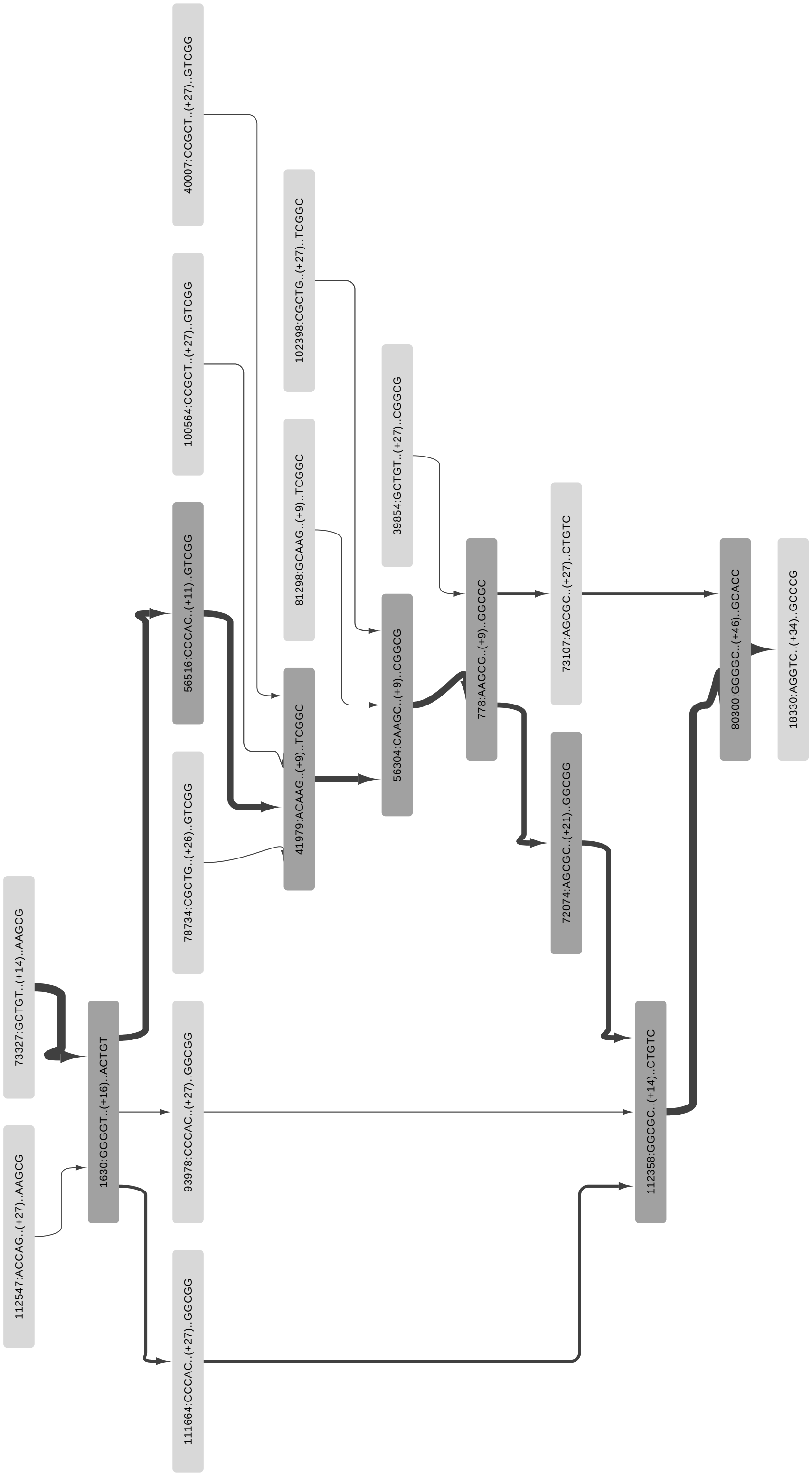

Figure S6 Cytoscape visualization of a subgraph of the pan-genome ccdBG of 341 *M. tuberculosis* strains ($k = 19$), corresponding to the end of the RRDR region of gene *rpoB*. Dark nodes represent the node path corresponding to the sequence for which visualization was requested.

Table S2 Overview of the number of alignments that are found by Nexus as well as BWA-MEM, but at a different edit distance. This table corresponds to the scatter plot in the right panel of Fig. 3 in the paper.

Nexus' edit distance	BWA-MEM's edit distance	Number of occurrences
1	2	3
2	3	235
2	4	57
2	5	10
2	6	11
2	8	1
3	4	544
3	5	117
3	6	44
3	7	1
3	8	12
3	12	1
3	23	9
4	5	792
4	6	349
4	7	64
4	8	1
4	9	1
4	12	6

Table S3 Extension of Tab. 10 in the paper for Bowtie 2 with different values for the `-k` parameter (the maximum number of alignments reported per read). The Bowtie 2 entry in Tab. 10 corresponds to Bowtie2 `-k` 10.

Tool	Avg. nr. of text occurrences/read	Avg. nr. of graph occurrences/read	Fraction of aligned reads	Performance		Peak RAM usage [GiB]
				[reads/s]	[text occs/s]	
Bowtie 2 -k 10	9.92	Not applicable	99.56%	2 591	25 705	40.08
Bowtie 2 -k 100	21.28	Not applicable	99.65%	131	2 779	40.09
Bowtie 2 -k 1000	115.95	Not applicable	99.72%	13	1 559	40.26

```
$ /nexus/build/visualizeRead -e 0 -d 1 -b -o outputFile
    ↪ MTuberculosisPanGenome 19
    ↪ ACCCACAAGCGCCGACTGTCGGCGCTGGGGCCCGGCGGTCTGTCA
```

The inputted DNA sequence corresponds to the last three codons from the RRDR region ("TCGGCGCTG"), padded with 18 nucleotides from the reference strain on either side, in order to visualize all nodes that contain a part of these three codons. The dark gray nodes in Fig. S6 correspond to the reference path in the manuscript's Fig. 5 (i.e., node path ADEFGHIK). Furthermore, nodes 111664, 93978 and 73107 in Fig. S6 correspond to respectively nodes B, C and J in Fig. 5 (manuscript), representing RRDR mutations S450L, S450W and L452P.

3 Reproducing Results

Following software versions were used for benchmarking:

- Nexus v1.1.0:
  https://github.com/biointec/nexus/releases/tag/v1.1.0
- Beller and Ohlebusch's A4 [7]:
  https://www.uni-ulm.de/in/theo/research/seqana.html

- deBGA [8] commit c2dbf6d6bb9bb27a0230b2d4022ec3e05efa46c9: https://github.com/HongzheGuo/deBGA/tree/c2dbf6d
- Pufferfish [9] (and PuffAligner [10]) v1.8.0: https://github.com/COMBINE-lab/pufferfish/releases/tag/salmon-v1.8.0
- (vg) Giraffe [11] v1.47.0 "Ostuni", specifically commit 80ee3fe: https://github.com/vgteam/vg/tree/80ee3fe
- BWA-MEM [12] v0.7.17: https://github.com/lh3/bwa/releases/tag/v0.7.17
- Bowtie 2 [13] v2.4.5: https://github.com/BenLangmead/bowtie2/releases/tag/v2.4.5

3.1 Obtaining the Reads

We sampled 100 000 reads from an Illumina experiment dataset (ftp://ftp.sra.ebi.ac.uk/vol1/fastq/ERR194/ERR194147/ERR194147_1.fastq.gz), which only contain 'A', 'C', 'G' and 'T' characters. The sampled dataset we used for the results in the paper is available on our GitHub page: https://github.com/biointec/nexus/releases/download/v1.0.0/sampled_illumina_reads.fastq. From now on, we refer to them as `reads.fastq`.

3.2 Commands for Building the Indexes (cf. Table 8)

3.2.1 deBGA construction

```
$ ./deBGA index -k 25 10HumanGenomes.fa deBGA/10HumanGenomes
```

3.2.2 Pufferfish construction

```
$ ./pufferfish index -r 10HumanGenomes.fa -o pufferfish/ --tmpdir
    ↪ pufferfishtemp/ -k 25 --noClip --filt-size 40 --threads 1
```

3.2.3 A4 construction

```
$ ./a4.x construct --inputfile=10HumanGenomes.fa --outputfile=a4
    ↪ /10HumanGenomes --kfile=kfile.txt
```

Where `kfile.txt` contains the value 25.

3.2.4 BWA construction

```
$ ./bwa index -p bwa/10HumanGenomes 10HumanGenomes.fa
```

A placeholder fasta header line needed to be added to each genome, since BWA cannot handle sequences longer than 2^{31} characters.

3.2.5 Bowtie 2 construction

```
$ ./bowtie2-build --offrate 4 --threads 1 --noauto --bmaxdivn 1 --
    ↪ dcv 16 10HumanGenomes.fa bowtie/10HumanGenomes
```

Where the parameters were chosen to make use of all available RAM, in order to optimize CPU time usage (within the limitations of 1 CPU thread).

3.2.6 Giraffe construction

```
$ ./vg autoindex --workflow giraffe --prefix giraffe/10
    ↪ HumanGenomes --ref-fasta 10HumanGenomes.fa --tmp-dir
    ↪ vgGiraffeKirlia/temp/ --verbosity 2 --threads 1
```

To be able to run this command, you must have the Giraffe version corresponding to the commit above (or higher). Otherwise, the graph cannot be built without the presence of a `.VCF` file. Additionally, a placeholder fasta header line needed to be added to each genome, since Giraffe cannot handle sequences longer than 2^{31} characters.

3.2.7 Nexus construction

```
$ ./nexusBuild -s 16 -c 128 -p 10HumanGenomes 25
```

Where input file `10HumanGenomes.txt` contains a preprocessed version of corresponding `10HumanGenomes.fa`. From each strain we removed all N's and substituted them with a random nucleotide. Then we removed the first line, concatenated the chromosomes, removed newline characters, and added a separation character to the end of file for each strain. Next, the strains can be concatenated into one pan-genome. An example of preprocessing a pan-genome of 3 strains is as follows:

```
$ tail -n +2 genome1.fasta.non | sed "s/>.*//g" | cat - percent |
    ↪ tr -d '\n' > genome1.txt
$ tail -n +2 genome2.fasta.non | sed "s/>.*//g" | cat - percent |
    ↪ tr -d '\n' > genome2.txt
$ tail -n +2 genome3.fasta.non | sed "s/>.*//g" | cat - dollar |
    ↪ tr -d '\n' > genome3.txt
$ cat genome1.txt genome2.txt genome3.txt > pangenome.txt
```

Where `genome1.fasta.non`, `genome2.fasta.non` and `genome3.fasta.non` are the result of the substitution of the N's for the input strains and `pangenome.txt` is the reference pan-genome used to build the index. `genome1.txt`, `genome2.txt` and `genome3.txt` are intermediate files which can be removed once the pan-genome is obtained. `dollar` and `percent` are text files containing a single character '$' and

'%', respectively. These files should be present in your directory before executing the above commands.

Since submitting the first version of the manuscript, Nexus v1.1.1 has become available, which is now able to create the index starting from `.fasta`/`.fa`/`.fna` file as well.

3.3 Command for Building All Nexus Indexes Used for Benchmarking

```
$ ./nexusBuild -s 1 -s 2 -s 4 -s 8 -s 16 -s 32 -s 64 -s 128 -s 256
    ↪  -c 8 -c 16 -c 32 -c 64 -c 128 -c 256 -c 512 -c 1024 -c 2048
    ↪  -c none -p 10HumanGenomes 25,50,75
```

3.4 Commands for Reproducing Table 9

3.4.1 A4

```
$ ./a4.x find_pattern --graphfile=10HumanGenomes.k25.bin --
    ↪ patternfile=reads.txt
```

Where `reads.txt` is a preprocessed text file containing only the DNA sequences contained in `reads.fastq`, no other information. Additionally, `reads.txt` also contains the reverse complement of all reads, as A4 does not match the reverse complements automatically.

3.4.2 Nexus

```
$ ./nexus -e <ED> -s 16 -c 128 -ss custom ../search_schemes/kuch_k
    ↪ +1_adapted/ 10HumanGenomes 25 reads.fastq
```

With `ED` ∈ {`0,1,2,3,4`}. Parameters:

- `-e`: maximum edit distance
- `-s`: suffix array sparseness factor
- `-c`: sparseness factor that indicates how many checkpoints must be stored to identify nodes
- `-ss`: search scheme

3.5 Commands for Reproducing Table 10

3.5.1 PuffAligner

```
$ ./pufferfish align -i pufferfish/ --read reads.fastq -t 1 -o
    ↪ output.sam --verbose --genomicReads
```

Where the `pufferfish/` directory contains the index. Parameters:

- `--genomicReads`: Align genomic dna-seq reads instead of RNA-seq reads

This command was also used for Fig. S2.

3.5.2 BWA-MEM

```
$ ./bwa mem -a -L 1000000,1000000 -Y -o bwa/output.sam bwa/10
    ↪ HumanGenomes reads.fastq
```

Where the `bwa/` directory contains the index. Parameters:

- `-a`: output all alignments instead of one per read
- `-L 1000000,1000000`: set a high penalty to avoid 5'- and 3'-end clipping
- `-Y`: if clipping is happens anyway, report all reads as soft clipped, not hard clipped

Even though we set a very high penalty to avoid clipping, a small fraction of occurrences still appears to be clipped. Since we are only interested in end-to-end alignment, we focused on non-clipped alignments for analyses such as in Fig. 3 in the paper. The non-clipped reads can be selected as follows:

```
$ grep -Ev "[0-9]+S" bwa/output.sam > bwa/output.notClipped.sam
```

3.5.3 Bowtie 2

```
$ ./bowtie2 --sensitive --end-to-end -k 10 --time --met-stderr -x
    ↪ bowtie/10HumanGenomes -U reads.fastq > bowtie/output.sam
```

Where the `bowtie/` directory contains the index. Parameters:

- `--end-to-end`: end-to-end alignment
- `--sensitive`: choose the parameter preset that performs sensitive alignment (i.e., prioritizing sensitity over performance to some degree)
- `-k`: maximum number of reported occurrences per read
- `--time` and `--met-stderr`: parameters for benchmarking

To generate Fig. S5, Bowtie 2 also run with `-k 100` and `-k 1000`.

3.5.4 Giraffe

```
$ ./vg giraffe -t 1 -Z giraffe/10HumanGenomes.giraffe.gbz -m
    ↪ giraffe/10HumanGenomes.min -d giraffe/10HumanGenomes.dist -f
    ↪  reads.fastq -o SAM -M 10
```

Where the `giraffe/` directory contains the index. Parameters:

- `-o`: output format of the alignments
- `-t`: number of mapping threads to use
- `-M`: maximum number of reported occurrences per read

Similar to BWA-MEM, Giraffe produces clipped alignments. Since we are only interested in end-to-end alignment, we focused on non-clipped alignments for analyses such as in Fig. S2. The non-clipped reads can be selected in the same way as in Section 3.5.2.

3.5.5 A4 and Nexus

See commands in Section 3.4.

3.6 Commands for Reproducing Figure 4

```
$ ./nexus -e 4 -s 16 -c <scp> -ss custom ../search_schemes/kuch_k
    ↪ +1_adapted/ 10HumanGenomes <k> reads.fastq
```

With `scp` ∈ {`8, 16, 32, 64, 128, 256, 512, 1024, 2048, none`} and `k` ∈ {`25,50,75`}.

3.7 Commands for Reproducing Figure S1

```
$ ./nexus -e 4 -s <sSA> -c 128 -ss custom ../search_schemes/kuch_k
    ↪ +1_adapted/ 10HumanGenomes 25 reads.fastq
```

With `sSA` ∈ {`1, 2, 4, 8, 16, 32, 64, 128, 256`}.

3.8 Case Study

3.8.1 Building the index

The preprocessed text file `MTuberculosisPanGenome.txt` containing the 341 concatenated *M. tuberculosis* strains can be found on our GitHub page: `https://github.com/biointec/nexus/releases/download/v1.1.0/MTuberculosisPanGenome.txt`.

Additionally, an annotation file is provide that contains the identifiers of these strains in the order in which they appear in the concatenated reference file: `https://github.com/biointec/nexus/releases/download/v1.1.0/MTuberculosisPanGenome.annotation.txt`.

Using the reference text file, the index can be built as follows:

```
$ ./nexusBuild -s 16 -c 128 -p MTuberculosisPanGenome 19
```

3.8.2 Reproducing the Case Study

To reproduce the case study, switch to the corresponding branch on GitHub: `https://github.com/biointec/nexus/tree/CaseStudy/casestudy`. Compile the code. Run the following command **inside the folder containing the index**.

```
$ ./casestudy
```

This executable searches for compensatory mutations as is described in the manuscript. Comments are provided in the `casestudy.cpp` script to outline the process. The case study results in a file `MTuberculosisPanGenome_Compensatory.tsv`, which contains the 14 candidate putative compensatory mutations that were also discussed in Table 12 in the manuscript. This file contains 5 fields:

- The RRDR mutation to which the candidate putative compensatory mutation corresponds;
- The identifier of the node in which the candidate putative compensatory mutation is found;
- The number of strains that carry the candidate putative compensatory mutation;
- The position of the candidate putative compensatory mutation with respect to the reference strain;
- The length of the node containing the candidate putative compensatory mutation.

Note again that the pipeline implemented here is more of an ad hoc solution to the problem of finding candidate compensatory mutations corresponding to mutations in the RRDR region of *rpoB*, rather than a general pipeline to be readily applied to other problems. For this reason, we provide the functionality in a `.cpp` file. If there were any interest to extend these ideas into a more general pipeline, possibly written in a more accessible script, do not hesitate to contact the authors of the manuscript.

Author details
[1]Department of Information Technology - IDLab, Ghent University - imec, Technologiepark 126, B-9052 Ghent (Zwijnaarde), Belgium. [2]Delft Bioinformatics Lab, Delft University of Technology, 2628 XE Delft, Netherlands. [3]Infectious Disease and Microbiome Program, Broad Institute of MIT and Harvard, MA 02142 Cambridge, USA.

References

1. Burrows, M., Wheeler, D.: A Block-Sorting Lossless Data Compression Algorithm. Research Report 124, Digital Equipment Corporation Systems Research Center, 130 Lytton Avenue, Palo Alto, California 94301 (May 1994)
2. Manber, U., Myers, G.: Suffix Arrays: A New Method for On-Line String Searches. SIAM Journal on Computing **22**(5), 935–948 (1993). doi:10.1137/0222058
3. Vigna, S.: Broadword Implementation of Rank/Select Queries. In: McGeoch, C.C. (ed.) Experimental Algorithms, pp. 154–168. Springer, Berlin, Heidelberg (2008). doi:10.1007/978-3-540-68552-4_12
4. Ferragina, P., Manzini, G.: Opportunistic data structures with applications. In: Proceedings 41st Annual Symposium on Foundations of Computer Science, pp. 390–398 (2000). doi:10.1109/SFCS.2000.892127
5. Lam, T.W., Li, R., Tam, A., Wong, S., Wu, E., Yiu, S.M.: High Throughput Short Read Alignment via Bi-directional BWT. In: 2009 IEEE International Conference on Bioinformatics and Biomedicine, pp. 31–36 (2009). doi:10.1109/BIBM.2009.42
6. Pockrandt, C., Ehrhardt, M., Reinert, K.: EPR-Dictionaries: A Practical and Fast Data Structure for Constant Time Searches in Unidirectional and Bidirectional FM Indices. In: Sahinalp, S.C. (ed.) Research in Computational Molecular Biology, pp. 190–206. Springer, Cham (2017). doi:10.1007/978-3-319-56970-3_12
7. Beller, T., Ohlebusch, E.: A representation of a compressed de Bruijn graph for pan-genome analysis that enables search. Algorithms for Molecular Biology **11**(1), 20 (2016). doi:10.1186/s13015-016-0083-7
8. Liu, B., Guo, H., Brudno, M., Wang, Y.: deBGA: read alignment with de Bruijn graph-based seed and extension. Bioinformatics **32**(21), 3224–3232 (2016). doi:10.1093/bioinformatics/btw371
9. Almodaresi, F., Sarkar, H., Srivastava, A., Patro, R.: A space and time-efficient index for the compacted colored de Bruijn graph. Bioinformatics **34**(13), 169–177 (2018). doi:10.1093/bioinformatics/bty292
10. Almodaresi, F., Zakeri, M., Patro, R.: PuffAligner: a fast, efficient and accurate aligner based on the Pufferfish index. Bioinformatics **37**(22), 4048–4055 (2021). doi:10.1093/bioinformatics/btab408
11. Sirén, J., Monlong, J., Chang, X., Novak, A.M., Eizenga, J.M., Markello, C., Sibbesen, J.A., Hickey, G., Chang, P.-C., Carroll, A., Gupta, N., Gabriel, S., Blackwell, T.W., Ratan, A., Taylor, K.D., Rich, S.S., Rotter, J.I., Haussler, D., Garrison, E., Paten, B.: Pangenomics enables genotyping of known structural variants in 5202 diverse genomes. Science **374**(6574), 8871 (2021). doi:10.1126/science.abg8871
12. Li, H.: Aligning sequence reads, clone sequences and assembly contigs with bwa-mem. arXiv preprint arXiv:1303.3997 (2013)
13. Langmead, B., Salzberg, S.L.: Fast gapped-read alignment with bowtie 2. Nature methods **9**(4), 357–359 (2012)
